# Ligand‐Mediated Surface Carrier Modulation in Perovskite Nanocrystals for Charge‐Symmetric LEDs

**DOI:** 10.1002/adma.202520499

**Published:** 2026-04-19

**Authors:** Jongho Park, Jisu Ha, Daehwan Kim, Hyeon Woo Kim, Han Uk Lee, Boseung Je, Dodam Kim, Jun‐Su Yeo, Min Gyo Kim, Changsoon Cho, Sung Beom Cho, Tae‐Hee Han

**Affiliations:** ^1^ Division of Materials Science and Engineering Hanyang University Seoul Republic of Korea; ^2^ Department of Materials Science and Engineering Ajou University Suwon Republic of Korea; ^3^ Department of Mechanical Engineering & Material Science Washington University in St. Louis St. Louis Missouri USA; ^4^ Department of Energy Systems Research Ajou University Suwon Republic of Korea; ^5^ Department of Materials Science and Engineering Pohang University of Science and Technology (POSTECH) Pohang Republic of Korea; ^6^ Institute for Convergence Research and Education in Advanced Technology Yonsei University Seoul Republic of Korea; ^7^ Department of Display Science and Engineering Hanyang University Seoul Republic of Korea

**Keywords:** ligand exchange, light‐emitting diodes, nanocrystals, perovskites

## Abstract

Metal halide perovskite nanocrystals (MHP NCs) hold great promise for light‐emitting diodes (LEDs), but face challenges due to weakly‐bound, insulating long‐chain ligands that impede charge transport and compromise stability. We introduce a hydrolysis‐assisted ligand‐exchange strategy that uses a multifunctional π‐conjugated pyridine carboxamide (PCA). This approach replaces native ligands to achieve strong surface binding and to strengthen electrical coupling, while imparting n‐type surface functionalization of MHP NCs. As a result, colloidal stability is improved, trap densities are reduced, and charge balance can be precisely tuned within LEDs. LEDs fabricated using ligand‐exchanged MHP NCs had a current efficiency of 121.4 cd A^−1^ and an external quantum efficiency of 31.7%, both of which are among the highest reported for green‐emitting LEDs that use MHP NCs. This study demonstrates the ligand‐exchange as a strategic approach for surface functionalization that enables control over charge balance and recombination dynamics, thereby significantly improving the efficiency of LEDs that use MHP NCs.

## Graphical abstract

A hydrolysis‐assisted ligand exchange strategy enables surface carrier modulation of perovskite nanocrystals using a multifunctional π‐conjugated ligand. This molecular surface design achieves charge balance and recombination‐zone symmetry in light‐emitting diodes (LEDs), which leads to high‐efficiency perovskite LEDs and establishes a general platform for surface modulation to optimize optoelectronic characteristics in nanomaterial‐based devices.

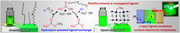

## 1 Introduction

Metal halide perovskites (MHPs) have exceptional optoelectronic properties and are therefore promising next‐generation light‐emitting materials [1, 2, 3, 4, 5, 6, 7, 8]. These properties include a narrow emission bandwidth (full width at half maximum, FWHM ≤ 20 nm) [2, 9, 10] and a broadly‐tunable bandgap covering the visible to near‐infrared spectrum [4, 9, 11, 12]. MHPs constitute the only class of light emitters that are capable of fully satisfying the Rec.2020 color gamut requirements, and are therefore promising resources to achieve advanced high‐definition displays that have high color purity [2].

However, practical application of colloidal MHP nanocrystals (NCs) in light‐emitting diodes (LEDs) is hindered by intrinsic limitations associated with surface chemistry and electrical charge‐transport properties [13, 14, 15]. The highly ionic nature and dynamic lattice structure of MHPs lead to inherently weak ligand‐surface interactions [16]. Moreover, reversible interactions between the cationic and anionic components of surface ligands readily allow their detachment from the NC surface [16, 17, 18, 19]. This ligand desorption readily results in formation of undercoordinated surface defect sites, particularly under electrical bias during device operation. Consequently, surface defects accumulate over time; this process accelerates device efficiency degradation and reduces device stability.

In addition, long‐chain insulating organic ligands severely disrupt electronic and electrical coupling between adjacent MHP NCs, and induce substantial accumulation of space charges within the NC emitting layer (EML) during device operation [20, 21]. The buildup of excited species accelerates charge‐induced degradation of MHP crystals, hinders high‐luminance emission, and leads to significant efficiency roll‐off (efficiency drop at high luminance) in LEDs [20].

To address these challenges, various ligand‐engineering strategies have been employed to replace native ligands with shorter or more robust alternatives [2, 7, 12, 13, 14, 15]. Although these approaches have improved charge transport between NCs, they often entail multiple harsh chemical treatment steps that degrade the properties of NCs [22]. These approaches typically involve the addition of excess long‐chain acids to disrupt the acid‐base equilibrium and remove native ligands, followed by introduction of target ligands [19]. However, this exchange process significantly compromises the colloidal stability and etches crystal surfaces, and these changes can reduce the structural integrity of the MHP NCs, altering their material properties [16, 17, 18, 19]. Furthermore, most ligand‐exchange approaches have focused primarily on improving charge transport; little attention has been given to tailoring the surface electronic structure to tune charge transport properties and recombination dynamics within the LED devices.

From a device perspective, achieving proper electron‐hole balance is critical for improving LED performance [23, 24, 25]. Imbalance in the transported carriers within the EML can lead to severe non‐radiative losses of charge carriers or excitons, and these losses both reduce luminous efficiency and accelerate device degradation by promoting trap formation and material decomposition [1, 26, 27]. Several strategies have aimed to improve charge injection by engineering the transport and injection layers of LEDs [28, 29], but comparatively little effort has been directed toward controlling the spatial position of the recombination zone by tuning the intrinsic electronic and interfacial properties of the MHP NC emitters themselves within the EML; this factor can be crucial for maximizing LED performance when the interfacial layers are well optimized.

Here, we introduce a multifunctional π‐conjugated pyridine carboxamide (PCA) for hydrolysis‐assisted one‐step ligand‐exchange of colloidal MHP NCs. This approach effectively removes weakly‐bound native ligands under mild conditions, and simultaneously introduces a chelating ligand that provides multidentate and multisite surface coordination and n‐type surface‐functionalization characteristics. By using PCA in this rationally‐designed ligand‐exchange strategy, we demonstrate that surface functionalization can be exploited not just for defect passivation, but also to directly influence charge balance and recombination dynamics in LED devices. This strategy leads to record‐high device efficiencies and provides a general framework for the surface design of NC or quantum dot emitters for optoelectronic applications.

## 2 Results and Discussion

### 2.1 Ligand Exchange of MHP NCs

The colloidal MHP NCs were synthesized using a ligand‐assisted reprecipitation method [9], which enables one‐step NC synthesis. The composition of MHP NCs was tuned to FA_0.816_GA_0.144_Cs_0.04_PbBr_3_, where the incorporation of oversized guanidinium ions (GA^+^) into the formamidinium (FA^+^)‐based MHP exceeds the substitutional doping limit. As a result, excess GA^+^ preferentially segregates toward the NC surface, leading to a surface‐segregated core‐shell‐like configuration consisting of an MHP crystal core and a GA‐rich surface layer [9, 20]. The energetic driving force and structural implications of this behavior are discussed in detail in the Figures S1–S5 and Note S1. A small amount of Cs^+^, which is smaller than FA^+^, was added to alleviate internal lattice microstrain within the MHP NC core.

**FIGURE 1:**
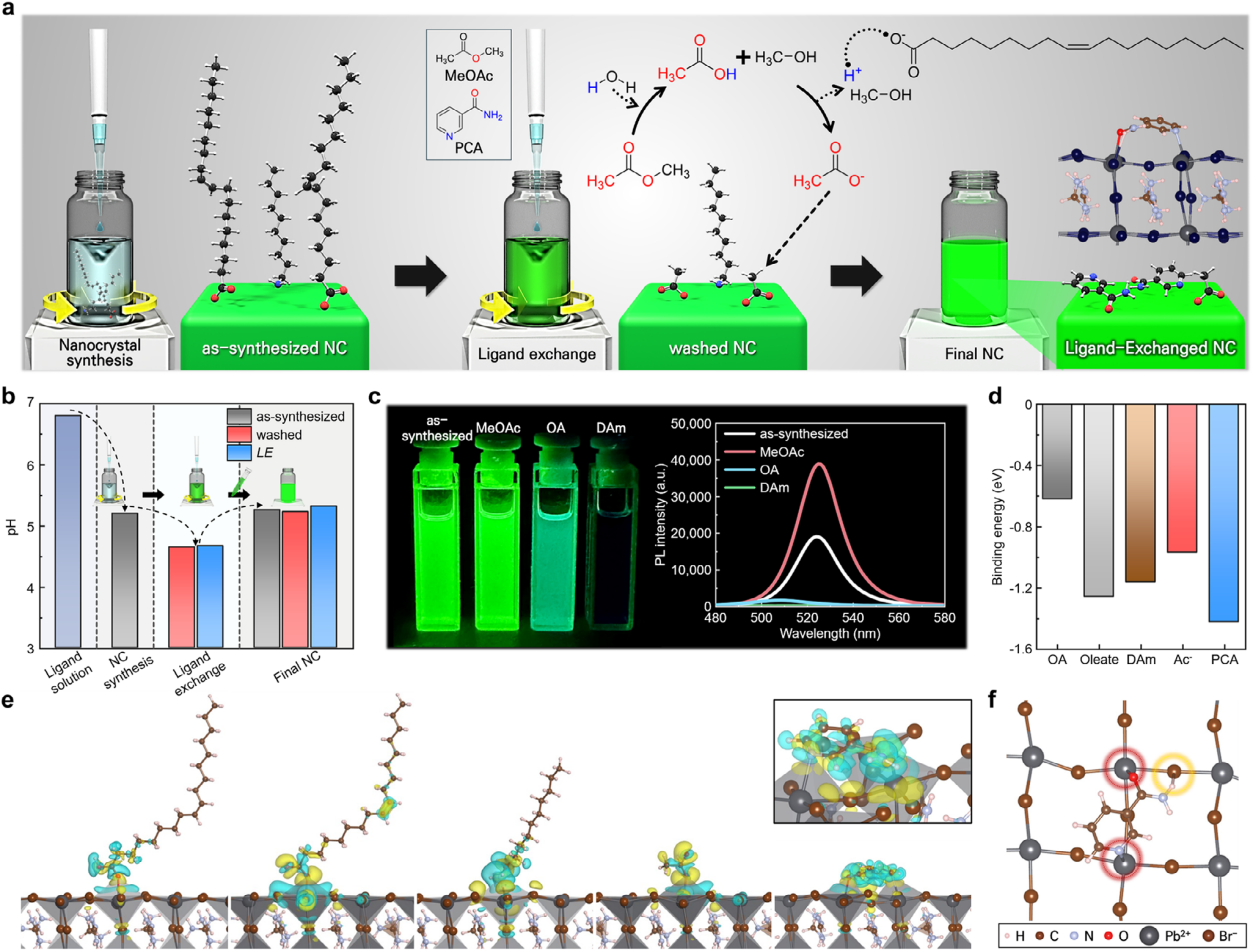
a) Schematic illustration of the ligand‐exchange and surface‐functionalization process of MHP NCs. b) pH values of ligand solutions, reaction mixtures, and final solutions of the as‐synthesized, washed, and *LE* NCs. c) Optical images of MHP NC solutions under 365 nm ultraviolet (UV) light illumination (left) and corresponding steady‐state PL spectra (right) without treatment and after treatment with MeOAc, OA, or DAm. d) Calculated binding energies of OA, oleate, DAm, Ac^−^, and PCA on the MHP NC surface. e) Optimized adsorption configurations of the ligands on the NC surface. f) Top view of the optimized *LE* NC surface coordinated with PCA.

The MHP NCs were initially synthesized with native long‐chain oleic acid (OA) and decylamine (DAm) ligands, which ensure colloidal stability during NC formation in solution. To enable functionalization of NCs, a novel hydrolysis‐assisted ligand‐exchange strategy was used to remove these native ligands and introduce multifunctional target ligands. This process allows functional target ligands to be introduced at the same time, and thereby enables both control over surface states, and tuning of NC properties. Methyl acetate (MeOAc) serves as a mild proton donor by in situ hydrolysis, which generates acetic acid and methanol (Figure 1a). The acetic acid protonates native oleate species, and thus enables their displacement from the NC surface. Simultaneously, deprotonation of acetic acid yields acetate (Ac^−^), which transiently coordinates to surface Pb^2^^+^ sites. When MeOAc and PCA are introduced together, this one‐step process facilitates effective removal of dominant OA and coordination of PCA to exposed undercoordinated sites on the NC surface (Figure 1a).

The conventional ligand‐exchange process typically involves stepwise addition of a ligand‐desorption agent, commonly excess OA, followed by introduction of a target ligand as a second step [19]. This exchange procedure relies on disrupting the existing acid‐base equilibrium by introducing excess acidic OA, which facilitates the detachment of native DAm from the MHP NC surface. However, this process frequently results in surface etching, which compromises the structural integrity and degrades surface characteristics of MHP NCs [19]. In contrast to conventional multi‐step processes, our ligand‐exchange strategy enables a single‐step replacement by co‐introducing proton‐donating MeOAc and target PCA ligand. This approach avoids excessive acid‐driven ligand desorption, thereby preventing surface etching and structural degradation, and offering a mild and effective route for ligand exchange and surface functionalization of MHP NCs (Figure 1a).

To validate the mechanism, we prepared three representative NC groups: (i) “as‐synthesized” NCs with native ligands, (ii) “washed” NCs treated with MeOAc only (Ac^−^‐coordinated), and (iii) “ligand‐exchanged (*LE*)” NCs treated with both MeOAc and PCA.

The pH of the reaction solution was monitored to validate the ligand‐exchange reaction (Figure 1b). The initial pH of the ligand solution that contained only OA and DAm was 6.8. After injection of the MHP precursors dissolved in dimethylformamide (DMF), the pH decreased to 5.2 due to the acidic nature of the precursor solution [30]. Addition of MeOAc alone or in combination with PCA to the pristine NC solution resulted in a further decrease in pH to approximately 4.7 (Figure 1b). This result provides direct evidence that MeOAc undergoes hydrolysis under the reaction conditions, and thereby generates protons that lead to the observed pH reduction. Also, the negligible difference between these two conditions indicates that PCA is chemically neutral and does not affect the exchange mechanism. After the purification process, the pH of the final solutions of the washed and *LE* NCs returned to 5.2 and 5.3, respectively; this result indicates that residual protons were effectively removed, and colloidal stability was restored.

Photoluminescence (PL) measurements showed that MeOAc treatment nearly doubled the PL intensity of as‐synthesized NCs; this response is consistent with transient surface passivation by Ac^−^ (Figure 1c). In contrast, conventional ligand‐desorption using excess OA or DAm led to significant reduction in PL intensity. The native ligands on as‐synthesized NCs were predominantly OA‐bearing (Figure S6 and Note S2), so removal of OA by DAm resulted in more extensive surface ligand loss and, consequently, a more severe decline in PL compared to those with OA treatment. The PL peak shift upon ligand treatment (Figure 1c) is primarily attributed to a surface etching‐induced reduction in effective NC size. In contrast to the hydrolysis‐based ligand exchange with MeOAc, which preserves both the NC size and emission energy, excess OA or DAm treatment induces a pronounced blue shift in the PL due to uncontrolled surface etching and crystal degradation (Figures S7 and S8). These results indicate that the deliberate ligand desorption coupled with transient passivation using Ac^−^ and target ligands increases the colloidal and photophysical stability of MHP NCs compared to the results of the conventional ligand‐desorption process (Figure S7) [19].

Density functional theory (DFT) calculations identified the binding energy *E*_b_ and the most stable binding configurations of ligands on the MHP NC surface (Figure 1d,e). Calculated *E*_b_ of OA on the MHP NC was −0.62 eV for the neutral form and −1.26 eV for the oleate form. Although the magnitude of *E*_b_ increases when OA is bound as oleate, subsequent MeOAc‐induced protonation converts oleate to its neutral form, thereby weakening the interaction and enabling effective removal of OA from the NC surface (Figure 1a).

The most stable surface binding configuration of PCA is a tri‐anchoring coordination on the NC surface (Figure 1e,f). In this configuration, DFT calculations revealed that the carbonyl and pyridine moieties directly coordinate to undercoordinated surface Pb^2+^ sites, and the amide concurrently interacts with a neighboring Br^−^ ion by a dipole‐mediated N─H···Br^−^ interaction. The tri‐anchoring coordination of PCA showed a significantly stronger *E*_b_ of −1.43 eV, and therefore a much more stable interaction than any single‐point coordination (Figure S9). Notably, this *E*_b_ also surpasses those of all native ligands and of the transient ligand Ac^−^; this comparison emphasizes PCA's ligand‐exchange effectiveness and strengthened binding stability after exchange (Figure 1d,e).

### 2.2 Surface Chemistry and Structural Analysis

**FIGURE 2:**
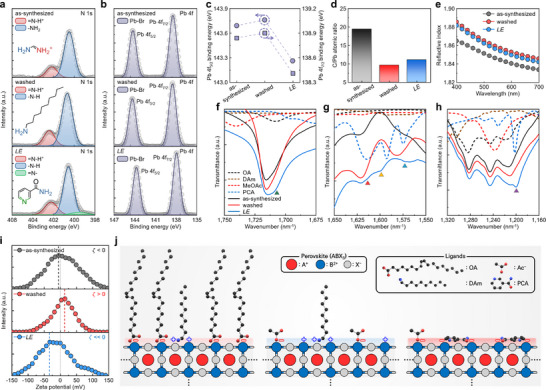
XPS spectra of a) N 1s and b) Pb 4f for the as‐synthesized, washed, and *LE* NCs. c) Binding energy shifts of Pb 4f peaks extracted from the fitted XPS spectra. d) Evolution of C to Pb ratio, and e) refractive index from 400 to 700 nm of the as‐synthesized, washed, and *LE* NCs. FTIR spectra of the ligands and MHP NCs for f) C═O stretching, g) N─H bending and C═N stretching, and h) C─N stretching. i) Zeta potential of NC surfaces for the as‐synthesized, washed, and *LE* NCs. j) Schematic illustration of MHP NC surface configurations.

X‐ray photoelectron spectroscopy (XPS) showed the elemental composition and chemical bonding states of the NC surfaces according to the three distinct stages (Figure 2a,b). The as‐synthesized and washed NCs had the same two peaks in the N 1s spectrum: one at ∼402.3 eV that is attributed to the protonated amine group (=N─H^+^) of formamidinium (FA^+^), and another at ∼400.5 eV that is attributed to neutral amine groups (─NH_2_) of FA^+^ and DAm. The *LE* NCs had an additional peak at ∼399.4 eV, which is attributed to the pyridinic nitrogen (=N─) on surface‐bound PCA, and confirms that it was successfully incorporated by the ligand‐exchange process (Figure 2a) [31].

The Pb 4f spectra showed peak shifts from 143.69 and 138.84 eV (as‐synthesized) to 143.76 and 138.90 eV (washed); these changes indicate decreased surface electron density due to the removal of OA and exposure of PbBr_2_‐terminated surfaces (Figure 2b,c). In the *LE* NCs, the *E*_b_ of Pb 4f peaks shifted to 143.26 and 138.41 eV; these changes suggest that surface electron density increased due to the coordination with PCA. Increased PCA concentration was correlated with a decrease in *E*_b_ of Pb‐Br; this trend indicates improved chelation and surface stabilization (Figure S10). This occurs because the pyridinic nitrogen and carbonyl oxygen in PCA chelate with Pb, donating electron density and suppressing electron withdrawal, which enhances core‐level screening and lowers the *E*_b_. Similar shifts of the Br 3d peak occurred; these changes further confirm strong surface coordination of PCA (Figures S11 and S12) [32].

Quantitative XPS analysis revealed a substantial decrease in the C/Pb ratio from 19.4 (as‐synthesized) to 9.6 (washed), which indicates effective removal of native long‐chain ligands and their partial replacement by short‐chain Ac^−^ ligands (Figure 2d). Subsequent ligand exchange with PCA increased the C/Pb ratio slightly to 11.1 due to the introduction of aromatic PCA, but the ratio remained well below that of the as‐synthesized NCs; this comparison is consistent with replacement of native ligands by shorter conjugated ligands (Figure S13). Spectroscopic ellipsometry also confirmed this trend, showing an increase in refractive index *n* from 1.847 (as‐synthesized) to 1.861 (washed), followed by a slight decrease to 1.858 (*LE*) at 550 nm; these changes are also consistent with reduced organic content in the NC film resulting from the ligand exchange (Figure 2e).

In Fourier transform infrared (FTIR) spectra, the *LE* NCs exhibited a distinct peak at 1710 cm^−1^, which corresponds to the C═O stretching vibration (Figure 2f and Figure S14a). This peak originates from the carbonyl group of PCA, and appears at 1698 cm^−1^ in free PCA [33]. The observed blueshift indicates strong coordination of the C═O carbonyl oxygen to surface Pb^2+^ of MHP NCs; this process increases both C═O bond order and vibrational frequency [34]. Additional PCA‐specific peaks appeared in the 1650–1550 cm^−1^ region (N─H bending, C═N stretching) (Figure 2g and Figure S14b), and a sharp C─N stretching peak at 1200 cm^−1^ intensified with increasing PCA (Figure 2h and Figure S14c); these observations collectively confirm effective surface binding of PCA on MHP NCs [35, 36].

Zeta potential measurements showed a near‐neutral value (−4.3 mV) for the as‐synthesized NCs due to the non‐polar nature of long‐chain ligands. After MeOAc treatment, zeta potential increased to +15.1 mV, as a result of partial exposure of positively‐charged surface Pb^2+^ sites. In the *LE* NCs, the zeta potential reversed to −31.9 mV, as a result of electron‐rich PCA ligands forming a negatively‐charged surface on MHP NCs (Figure 2i,j) [37].

**FIGURE 3:**
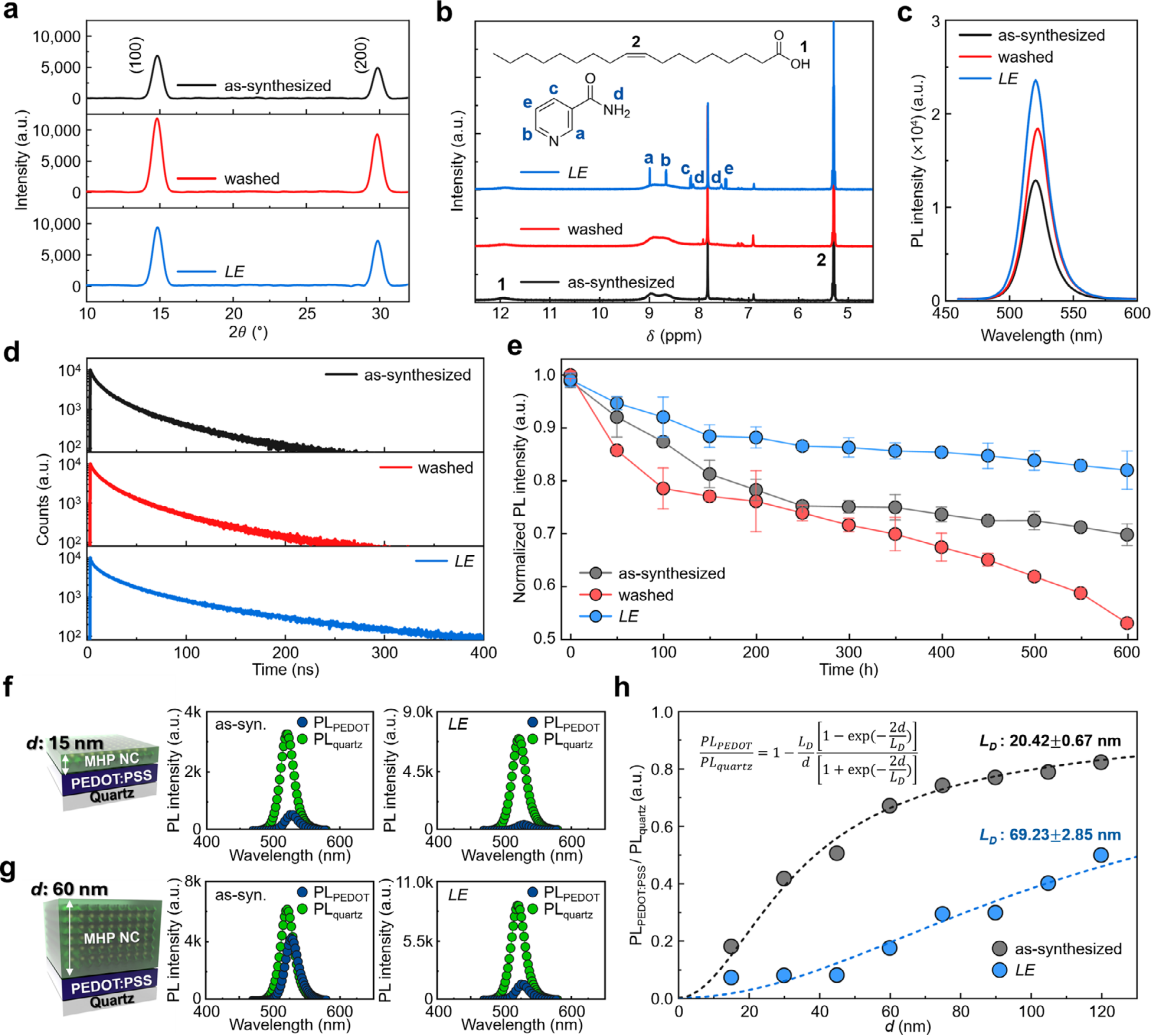
a) XRD and b) ^1^H NMR spectra of the as‐synthesized, washed, and *LE* NCs. c) Steady‐state PL spectra, and d) TRPL decay curves of the corresponding NC films. e) Time evolution of steady‐state PL intensity under ambient conditions at room temperature. PL intensity of the as‐synthesized (left) and *LE* NC films (right) on PEDOT:PSS or quartz substrates with NC EML thickness of f) 15 and g) 60 nm. h) PL intensity of the as‐synthesized and *LE* NC films on PEDOT:PSS normalized to that on quartz as a function of EML thickness.

Transmission electron microscopy (TEM) analysis indicated that the inter‐NC distance decreased substantially from ∼2.47 nm (as‐synthesized) to ∼1.75 nm (washed) to ∼0.98 nm (*LE*) (Figures S15 and S16); this trend indicates progressive removal of bulky ligands and tightening of packing due to compact tri‐anchoring PCA coordination. In addition, the average NC diameter increased from 8.68 nm (as‐synthesized) to 9.72 nm (washed) (Figure S17), likely due to surface growth driven by precursor diffusion after native ligand removal. The X‐ray diffraction (XRD) spectra showed peaks at 2θ = 14.8° and 29.9^°^, which correspond respectively to the (100) and (200) of FA_0.816_GA_0.144_Cs_0.04_PbBr_3_. These peak intensities increased in the washed NCs due to their increased crystallite size (Figure 3a). Upon ligand exchange with PCA, the average NC diameter was 9.62 nm, which is slightly smaller than that of washed NCs (Figure S17), accompanied by a slight reduction in XRD peak intensity (Figure 3a). This is attributed to the formation of a rigid and strongly‐bound surface ligand shell, which suppresses further precursor diffusion and additional surface crystal growth [20].

The ligand exchange mechanism was further elucidated via systematic ^1^H nuclear magnetic resonance (NMR) analysis (Figure 3b and Figures S18–S20 and Note S3). Upon MeOAc washing, native oleate ligands were effectively removed via acid‐mediated hydrolysis, as evidenced by a recovered proton integration ratio matching free OA (Figures S18 and S19). After PCA treatment, characteristic aromatic proton signals (7–9 ppm) appeared and remained after purification, confirming stable adsorption of PCA on the NC surface (Figures S18 and S19). Importantly, PCA binding occurred without further displacement of OA, and the PCA:OA ratio increased with input concentration (Figure S19), supporting efficient and tunable surface coordination.

Together, these analyses confirm that PCA ligands effectively bind to the MHP NC surfaces through multidentate and multisite coordination, replacing the long‐chain native ligands and forming compact, electronically active ligand shells that stabilize the NCs both structurally and electronically.

### 2.3 Photophysical Properties of Ligand‐Exchanged MHP NCs

Upon strong binding of PCA to the MHP NC surface by multi‐anchoring coordination, the steady‐state PL intensity nearly doubled compared to that of the as‐synthesized NC film (Figure 3c and Figures S21 and S22). Time‐resolved PL (TRPL) decay was fitted using a bi‐exponential model. Following surface passivation by strongly‐bound PCA, the fast‐decaying component τ_1_ in TRPL increased substantially from 4.1 ns (as‐synthesized) to 7.6 ns (*LE*), while the proportion of τ_1_ component *A*_1_ decreased from 63.4% to 55.4%. In parallel, the slow‐decaying component τ_2_ increased from 23.6 ns (as‐synthesized) to 32.6 ns (*LE*), with the corresponding proportion *A*_2_ increasing from 36.6% to 44.6%; these changes indicate suppressed nonradiative recombination due to the increased passivation of surface defects. As a result, the average PL lifetime τ_*avg*_ increased from 11.3 ns (as‐synthesized) to 18.8 ns (*LE*), along with increased PL quantum yield (Figure 3d and Figures S23–S25 and Tables S1 and S2).

Colloidal stability of each NC solution was also evaluated by monitoring the PL over 600 h of storage. The washed NCs retained only 53% of initial value PL_0_, and the as‐synthesized NCs retained approximately 69% of PL_0_, but the *LE* NCs retained over 82% of PL_0_, which demonstrates a markedly improved colloidal stability of *LE* NCs (Figure 3e). The low colloidal stability in washed NCs can be attributed to insufficient surface passivation and diminished steric repulsion, which together promote interparticle aggregation. The increased stability of *LE* NCs arises from the strong multidentate and multisite coordination of PCA, which passivates surface defects, suppress ligand desorption, and inhibit aggregation over time.

Water contact angle measurements provided further evidence of the surface modification of NCs. The *LE* NC films had a static contact angle of 27°, which is significantly higher than that of the as‐synthesized and washed NCs (both ∼9°); the difference indicates a decrease in hydrophilicity of the *LE* NC films. This behavior can be attributed to the PCA‐induced multi‐anchoring configuration, in which PCA coordinates laterally to the NC surface through multisite interactions, forming a compact ligand shell that efficiently screens polar ionic surface terminations. This result supports the hypothesis that multi‐anchoring coordination of PCA leads to a compact and less‐hydrophilic ligand shell and a decrease in the number of surface defects on the *LE* NCs (Figure S26 and Note S4).

### 2.4 Electronic and Electrical Modulation of MHP NCs

To evaluate whether ligand exchange promotes interparticle electronic coupling, we measured the exciton diffusion length (*L*_D_) of the NC films using an exciton‐quenching model based on differences in PL intensity between non‐quenching (quartz) and quenching (PEDOT:PSS) substrates (Figure S27 and Note S5) [24]. The *LE* NC films exhibited relatively large PL differences (PL_quartz_‐PL_PEDOT:PSS_) with minimal thickness dependence, whereas the as‐synthesized NC films showed a rapid decrease in PL difference with increasing thickness, indicating limited exciton diffusion (Figure 3f,g). By fitting the normalized PL ratio to a one‐dimensional diffusion model (Figure 3h) [24], we determined that the *LE* NCs have a significantly longer *L*_D_ of 69.2 ± 2.85 nm compared to 20.4  ±  0.67 nm for the as‐synthesized NCs. This enhancement is attributed to PCA's π‐conjugation, short molecular length, and multidentate binding, which facilitate wavefunction delocalization and stronger inter‐NC electronic coupling.

**FIGURE 4:**
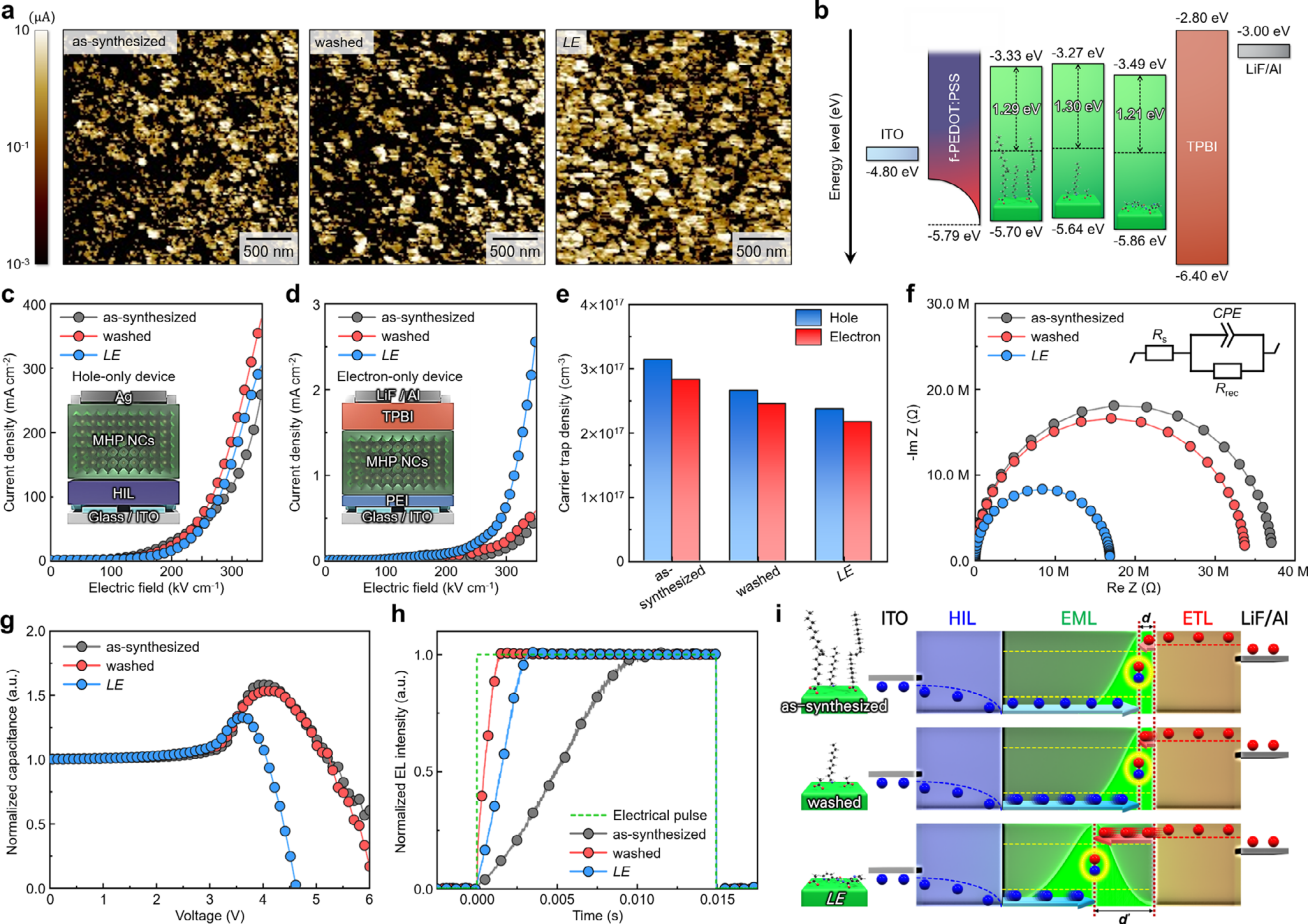
a) c‐AFM electrical current mapping of the as‐synthesized, washed, and *LE* NCs. b) Flat‐band energy level diagram of MHP NC‐based LEDs using as‐synthesized (left), washed (middle), and *LE* (right) NCs as an EML. Current density vs electric field characteristics of c) hole‐only devices (HODs) (inset: illustration of fabricated HOD architecture) and d) electron‐only devices (EODs) (inset: illustration of fabricated EOD architecture). e) Hole and electron trap densities extracted from the trap‐filled limit regime of HODs and EODs. f) Nyquist plots measured at 0 V under dark conditions, and g) normalized capacitance vs voltage characteristics of MHP NC‐based LEDs. h) Transient EL characteristics, and i) schematic illustration of charge carrier transport properties and recombination zone positions of MHP NC‐based LEDs with the as‐synthesized, washed, and *LE* NCs.

Conductive atomic force microscopy (c‐AFM) mapping was used to visualize the nanoscale distribution of electrical properties across the NC films that had different surface ligands (Figure 4a). The as‐synthesized NCs had sparse, localized current paths, and the washed NCs showed moderately improved conduction due to reduced long‐chain ligand barriers, but the *LE* NCs demonstrated the most uniform and highest conduction; this is attributed to compact packing and π‐conjugated pyridine moiety of PCA, which promotes surface electron delocalization [33, 38]. This trend is evident in the average current values, which increased to 442 nA in the *LE* NC films from 213 nA in the as‐synthesized NC films. The electron‐rich nature of PCA also contributes to n‐type surface functionalization, which increases the number of free electron carriers and facilitates electron transport across the film.

We investigated how the strong surface coordination of PCA influences the energy levels of NCs. In ultraviolet photoelectron spectroscopy (UPS) analysis, the Fermi level *E*_F_ shifted toward the conduction band minimum (CBM) after PCA coordination on the NC surface, with the energy gap between CBM and *E*_F_ decreasing from 1.29 to 1.21 eV (Figure 4b and Figures S28–S30 and Note S6). This shift can be attributed to surface dipole formation, in which the outward‐oriented, electron‐rich pyridine group of PCA modulates the interfacial electrostatic potential and shifts the vacuum level. The reduction of CBM‐*E*_F_ indicates an increase in the concentration of free electrons in MHP NCs [39]. This n‐type doping behavior can be attributed to the electron‐donating nature of the pyridine units in PCA (Figure 1e), which transfer electron density to the MHP NC surface. This increase in surface electron density leads to an increase in the efficiency of electron transport across the NC film, and thus contributes to the improved electron‐conductive properties that occur after ligand exchange.

To investigate how surface functionalization influences charge carrier transport in the MHP NCs, single‐carrier current densities were assessed using hole‐only devices (HODs) and electron‐only devices (EODs). The HODs were fabricated with the structure ITO/self‐organized surface‐fluorinated PEDOT:PSS (*f*‐PEDOT:PSS)/MHP NC EML/Ag (Figure 4c). The *f*‐PEDOT:PSS is composed of poly(3,4‐ethylenedioxythiophene):poly(styrenesulfonate) (PEDOT:PSS) and perfluorinated sulfonic acid (PFSA). The surface‐energy difference between these polymeric components induces self‐organization, which gradually increases the ionization potential (IP) toward the surface to ∼5.79 eV and effectively reduces the energy barrier for hole injection into the MHP NC EML [24, 40].

The EODs were fabricated with the structure ITO/polyethylenimine (PEI)/MHP NC EML/2,2′,2′′‐(1,3,5‐Benzinetriyl)‐tris(1‐phenyl‐1‐*H*‐benzimidazole) (TPBI)/LiF/Al (Figure 4d). The thin PEI layer induces surface dipole alignment, which causes a vacuum‐level shift that lowers the surface work function of the ITO [41]. This shift suppresses hole injection from the ITO and facilitates electron‐dominant transport in EODs [42].

In the HODs, the hole current density (*J*_p_) increased slightly after ligand washing and exchange, mainly due to the removal of insulating long‐chain ligands, which lowers the dielectric barrier for inter‐NC hole transport. After PCA exchange, *J*_p_ decreased marginally compared to washed NCs, which is attributed to a slight increase in surface carbon content originating from surface‐bound PCA molecules (Figure 4c).

In contrast, the EODs exhibited a pronounced increase in electron current density (*J*_n_) for the PCA‐treated NCs, demonstrating that PCA treatment preferentially enhances electron transport rather than hole transport. This behavior is consistent with the π‐conjugated and electron‐rich nature of PCA, which promotes surface‐electron delocalization and facilitates inter‐NC electron transport (Figure 4d) [38].

From the trap‐filled limit (TFL) regime, the charge trap densities *n_t_* s were calculated using the following equation:

(1) $$n_{t}=\frac{2V_{TFL}\varepsilon_{0}\varepsilon_{r}}{eL^{2}}$$

where *V*_TFL_ is the TFL voltage, ε_0_ is the vacuum permittivity, ε_*r*_ is the relative permittivity (for FAPbBr_3_, ε_*r*__=_ 43.6) [43], *e* is the electron charge and *L* is the thickness of the MHP NC films. The calculated trap densities decreased both for holes (from 3.14 × 10^17^ to 2.37 × 10^17^ cm^−3^) and electrons (from 2.83 × 10^17^ to 2.17 × 10^17^ cm^−3^) after ligand exchange with PCA (Figure 4e and Figure S31). These results indicate that ligand washing and subsequent exchange with PCA effectively passivate surface trap states for both holes and electrons, thereby improving the overall optoelectronic properties of NC films.

### 2.5 Charge Carrier Dynamics in Light‐Emitting Diodes

We then evaluated how the observed improvements in charge‐carrier transport and trap passivation translated into device performance in LEDs that use the surface‐functionalized NCs. The LEDs had the configuration ITO/*f*‐PEDOT:PSS/MHP NC EML/TPBI/LiF/Al (Figure 4b). Electrochemical impedance spectroscopy was used to investigate the charge injection and recombination dynamics of the LEDs. Nyquist impedance spectra showed semicircular arcs in the low‐frequency region, corresponding to recombination resistance *R*_rec_ (Figure 4f) [44]. The arc radius decreased from as‐synthesized to washed and *LE* devices; this trend indicates a progressive decrease in charge accumulation, and is consistent with the reduced trap density and improved charge transport arising from effective ligand replacement and surface functionalization with PCA.

In analysis of capacitance–voltage *C*–*V* characteristics, a sharp increase in capacitance at low voltage indicates injection and accumulation of majority charge carriers. As the voltage increases, these accumulated majority carriers begin to recombine with injected minority carriers, so the capacitance declines [1, 26, 27]. The *LE* NC device showed the lowest peak capacitance at the lowest voltage; this result suggests that this device has the earliest onset of minority‐carrier injection and transport, along with the most balanced electron‐hole transport (Figure 4g). Furthermore, the *LE* device showed the steepest decrease in capacitance in the high‐voltage regime; this result indicates more efficient recombination and minimal charge buildup during operation compared to the other devices.

Transient electroluminescence (TREL) of LEDs was also conducted to directly assess how the altered charge‐transport properties of NCs influence charge symmetry and the position of the recombination zone in the LEDs (Figure 4h). Comparison of the TREL profiles of LEDs that used conventional PEDOT:PSS and efficient hole‐injecting *f*‐PEDOT:PSS revealed that holes are the dominant majority carriers, and that the recombination zone is likely to be located near the EML/electron transport layer (ETL) interface, where the injected electrons encounter accumulated holes, in our LED structure (Figure 4i and Figure S32 and Note S7). The device incorporating the washed NCs exhibited a significantly shorter EL rising time (*t_r_* defined as the time to reach 80% saturation intensity: ∼0.88 ms) compared to that with the as‐synthesized NCs (∼7.42 ms), whereas the *LE* NC device showed a slightly slower EL rise (*t_r_*: ∼2.32 ms) than the washed NC device (Figure 4h).

In the washed NCs, replacement of long‐chain insulating ligands with short‐chain Ac^−^ reduces the tunneling barrier and dielectric thickness at the NC surface, thereby increasing the efficiency of charge‐carrier injection and transport between NCs. As in single‐carrier devices, this washing process increases both hole and electron transport concurrently. Therefore, the recombination zone likely remains in a similar position to that in the as‐synthesized NC device (Figure 4i). However, the overall improvement in carrier transport decreases the *t_r_* (Figure 4h). The π‐conjugated pyridine ring induces n‐type functionalization at the NC surface and promotes surface charge delocalization. This change improves electron transport in the EML and consequently improves carrier‐transport balance, and thus increases the distance that minority carriers (i.e., electrons) should travel from cathode to recombination zone in EML, so *t_r_* is slightly longer than in the washed NCs [26, 27, 45, 46]. The improved balance in carrier transport shifts the recombination zone toward the center of the EML from the interface and broadens recombination profile within the EML (Figure 4i); consequently, the recombination zone moves away from the heterointerfaces in LEDs; this change effectively suppresses field‐assisted exciton dissociation and exciton annihilation, and thereby increases the efficiency of the radiative recombination process [26, 27, 47].

Improved inter‐NC electronic coupling alone is insufficient for achieving high device efficiency if excitons are quenched at the HIL/NC interface. To address this, we employed a self‐organized surface‐fluorinated HIL that forms a molecular separation barrier. This layer effectively suppresses interfacial quenching while improving hole injection efficiency [24]. The combination of ligand‐driven charge and exciton delocalization with enhanced interfacial robustness is essential for realizing the high luminous efficiency observed in our NC‐based LEDs.

**FIGURE 5:**
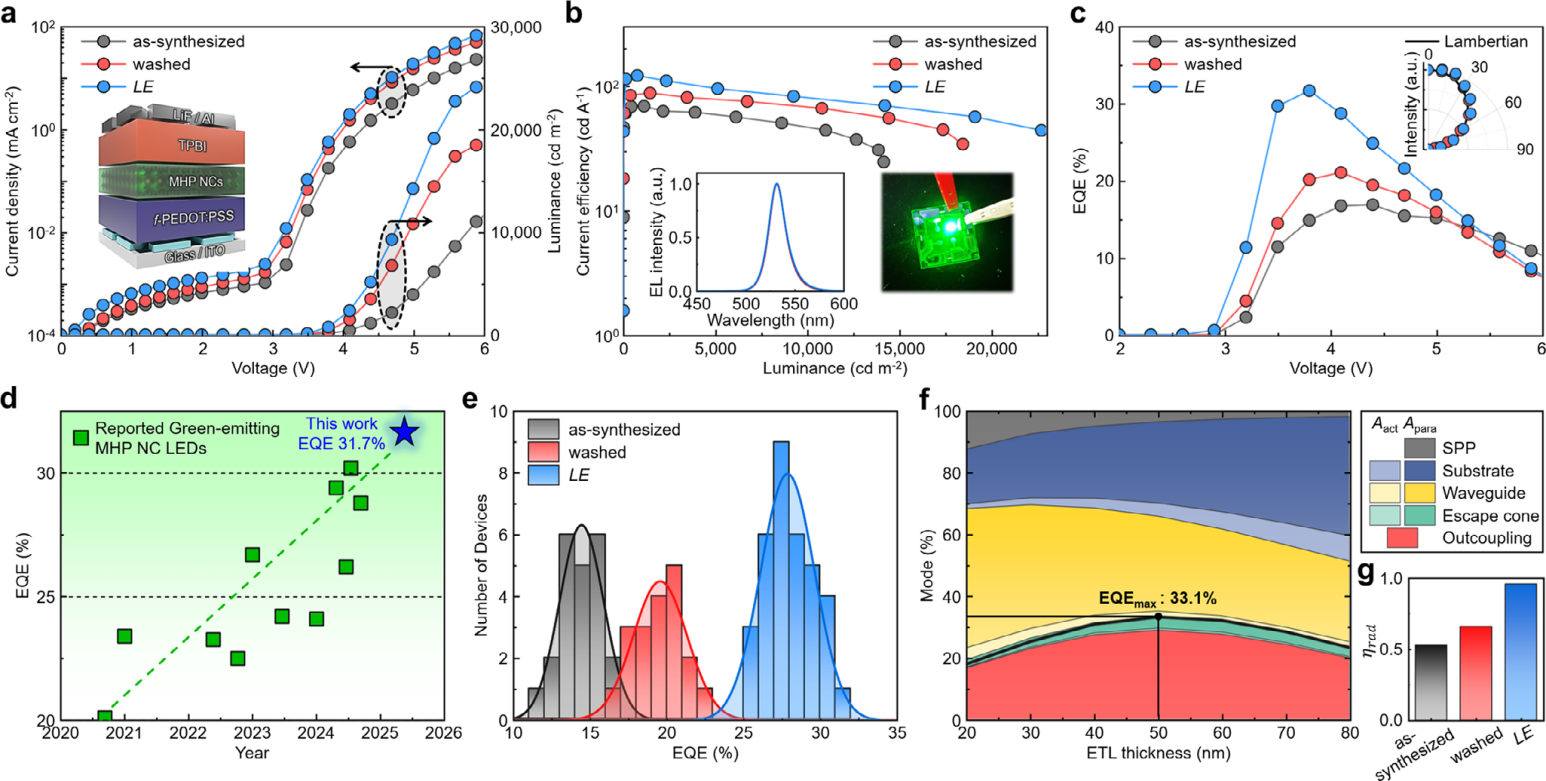
a) Current density and luminance vs voltage characteristics (inset: illustration of fabricated LED device architecture), b) current efficiency vs luminance characteristics (inset: EL spectrum (left) and optical image of the *LE* NC‐based LED lighting (right)), c) EQE vs voltage characteristics (inset: viewing angle‐dependent EL emission profile used for EQE calculation) of MHP NC LEDs. d) Comparison of reported EQE values of green‐emitting MHP NC‐based LEDs from 2020 to present. e) EQE histogram of LEDs that use the as‐synthesized, washed, or *LE* NCs as an EML. f) Optical simulation of relative mode fraction in *f*‐PEDOT:PSS‐based MHP NC LEDs as a function of electron transport layer (ETL) thickness, and g) internal radiation efficiency η_*rad*_ of the as‐synthesized, washed, and *LE* NCs.

The LED device performance metrics are strongly affected by the improvements in charge symmetry. The *LE* NC LEDs exhibited higher current density and luminance compared to the as‐synthesized NC LEDs (Figure 5a). The maximum luminance was ∼25000 cd m^−2^ in the *LE* NC LEDs, compared to ∼13000 cd m^−2^ for the as‐synthesized NC LEDs. The maximum current efficiency of *LE* NC LEDs was 121.4 cd A^−1^, which significantly exceeds the 68.7 cd A^−1^ of as‐synthesized NC LEDs and 87.7 cd A^−1^ of washed NC LEDs; this comparison demonstrates that the surface ligand engineering increased both the charge transport symmetry and the recombination efficiency (Figure 5b). The EL spectrum of *LE* NC device had a narrow FWHM ∼20 nm centered at 532 nm (inset in Figure 5b). The external quantum efficiency (EQE), calculated from each angular EL profile (inset in Figure 5c) reached 31.7%; to the best of our knowledge, this is the highest luminous efficiency reported for LEDs that use MHP NCs (Figure 5c,d) [5, 9, 48, 49, 50, 51, 52, 53, 54, 55, 56]. These improvements in device characteristics were maximized at an optimized PCA concentration, where charge balance and recombination efficiency were simultaneously improved (Figure S33). Although some efficiency roll‐off remains at high luminance, this behavior is mainly attributed to high‐density recombination processes under strong carrier injection, while *C–V* and TREL analyses (Figure 4c–i) confirm that PCA treatment substantially suppresses charge accumulation and improves recombination balance [57, 58, 59]. The EQE histogram of *LE* NC devices further demonstrated high effectiveness and excellent device‐to‐device reproducibility (Figure 5e).

To validate the molecular design strategy, we systematically compared PCA with control ligands that differ in acidity and conjugation (Figures S34–S37 and Table S3 and Note S8). Only PCA, featuring neutrality and heteroaromatic pyridine moiety, provided significant improvements in photophysics and device characteristics, confirming the unique role of PCA.

To decouple the role of A‐site composition from that of ligand exchange, we systematically compared LED devices based on three representative NCs with identical PCA treatment: single‐cation (FA, FAPbBr_3_), binary (FA‐GA, FA_0.815_GA_0.185_PbBr_3_), and ternary (FA‐GA‐Cs, FA_0.816_GA_0.144_Cs_0.04_PbBr_3_) systems. Stepwise improvements in PLQY, reduction in trap densities, and increases in average EQEs were observed, confirming the progressive benefits of A‐site engineering on luminous characteristics (Figures S38–S41 and Note S9).

To confirm that the observed enhancements are primarily driven by the PCA ligand exchange rather than A‐site composition effects, we further applied our strategy to MHP NCs with fully inorganic (CsPbBr_3_) and single organic A‐site compositions (FAPbBr_3_ and MAPbBr_3_). Across all cases, PCA binding was spectroscopically verified, and consistent improvements in PL, carrier balance, and LED characteristics were observed after ligand exchange (Figures S42–S48 and Tables S4 and S5 and Note S10). These results confirm the general applicability of our surface coordination approach across diverse MHP systems.

To quantify the optical efficiencies of our device, we calculated the fractions of direct outcoupling, perovskite reabsorption *A*_act_, and parasitic absorption loss *A*_para_ at each optical mode [60, 61]. The maximum EQE of *LE* NC LEDs was calculated to be 33.1%, slightly above the fraction of direct outcoupling 30.9%, due to photon recycling that additionally extracts the reabsorbed photons (*A*_act_) in the trapped modes (Figure 5f) [60, 61]. The calculations also revealed that the EQE_max_ increased from 31.0% with the PEDOT:PSS to 33.1% with the *f*‐PEDOT:PSS (Figure S49). The reduced *n* of *f*‐PEDOT:PSS leads to a more favorable mode distribution, decreasing substrate‐mode losses and improving light extraction into radiative channels, thereby increasing overall light outcoupling of the MHP NC LEDs [62]. These results indicate that the measured EQE of 31.7% for our *f*‐PEDOT:PSS‐based *LE* NC LEDs approaches the simulated optical outcoupling limit, confirming the near‐unity internal radiative efficiency η_*rad*_ of the *LE* NC LEDs and the optimized *n* profile of the interfacial layer in the device. A comparison of the η_*rad*_ across different MHP NC LEDs showed a significant enhancement from 0.53 in the as‐synthesized NCs to 0.66 in the washed NCs and further to 0.96 in the *LE* NCs (Figure 5g). These results confirm that ligand exchange using PCA is an effective method to optimize charge balance, suppress interfacial losses, and achieve record‐setting performance metrics in MHP NC LEDs.

In addition to high efficiency, the *LE* NC LEDs also exhibited markedly improved operational stability (Figure S50 and Note S11). Under constant current operation, the device half‐lifetime *LT*_50_ was enhanced by more than an order of magnitude compared to the as‐synthesized reference. This improvement can be attributed to suppressed charge accumulation and enhanced interfacial robustness arising from the PCA‐induced charge delocalization and improved surface passivation. Compared with 3D polycrystalline MHP LEDs, the shorter operational stability typically observed in NC LEDs mainly originates from the intrinsic architecture of NC solids, where charge transport and recombination involve numerous NC–NC interfaces even when the surface is effectively passivated [2, 20].

## 3 Conclusion

We developed a one‐step ligand‐exchange strategy using a multi‐anchoring and π‐conjugated PCA in MHP NCs to simultaneously achieve strong surface binding, strengthened electrical coupling, and n‐type surface functionalization. The novel hydrolysis‐assisted transient capping strategy effectively replaced long‐chain insulating native ligands while preserving colloidal stability and preventing structural degradation that conventional exchange protocols can cause. This ligand‐exchange approach thereby enables improved surface passivation and stable colloidal dispersions while minimizing structural damage.

Surface analyses and DFT calculations confirmed the successful tri‐anchoring coordination of PCA and the resulting n‐type surface doping behavior. Electrical measurements from single‐carrier devices, trap analysis, and UPS collectively demonstrated that ligand exchange with PCA predominantly increases electron transport and passivates surface trap states. As a result, the recombination zone in LEDs incorporating *LE* NCs shifted toward the center of the EML, thereby reducing interfacial losses and increasing radiative recombination efficiency.

The resulting *LE* NC LEDs achieved a peak luminance of ∼25000 cd m^−2^, a current efficiency of 121.4 cd A^−1^, and an EQE of 31.7%, which is among the highest reported values for MHP NC LEDs. This work emphasizes the effectiveness of surface ligand design in modulating the electrical and optoelectronic properties of MHP NCs, and provides an effective method to produce highly‐luminous and highly‐efficient charge‐symmetric MHP LEDs.

This strategy provides a general approach for surface design of NC emitters for optoelectronic applications. Furthermore, the ligand‐exchange method demonstrated here can be applied to other MHP compositions and devices, and to increase the performance in diverse optoelectronic systems.

## 4 Experimental Methods

### 4.1 Materials

FABr (99.99%) and GABr (99.99%) were purchased from Greatcell Solar. CsBr (99.999% metal basis), and PbBr_2_ (98% metal basis) were purchased from Alfa Aesar. DMF (anhydrous, ≥99.9%), toluene (anhydrous, 99.8%), 1‐butanol (anhydrous, 99.8%), oleic acid (90%), decylamine (≥99.0%), methyl acetate (anhydrous, 99.5%), pyridine carboxamide (≥99.5%), tetrafluoroethylene‐perfluoro‐3,6‐dioxa‐4‐methyl‐7‐octene‐sulfonic acid copolymer, lithium fluoride (>99.99%), and branched polyethylenimine were purchased from Sigma‐Aldrich. PEDOT:PSS (CLEVIOS P VP AI4083) was purchased from Heraeus. Acetone (>99.5%) and isopropanol (>99.5%) were purchased from Daejung Chemicals. 2,2′,2″‐(1,3,5‐Benzinetriyl)‐tris(1‐phenyl‐1‐*H*‐benzimidazole) was purchased from OSM. Al and Ag were purchased from iTASCO.

### 4.2 Synthesis of Colloidal MHP NCs

The colloidal MHP NCs were synthesized under ambient conditions. The precursor solution was prepared by dissolving FABr, GABr, CsBr, and PbBr_2_ in DMF at appropriate molar ratios. First, 225 µL of the precursor solution was injected into a crystallization‐inducing solvent mixture containing 5.0 mL of toluene, 2.0 mL of 1‐butanol, 0.3 mL of OA and 25 µL of DAm. The mixture was stirred vigorously for 9 min., and then purified by sequential centrifugation and redispersed in toluene. For the washed and *LE* NCs, an additional ligand‐exchange step was introduced. After 9‐min. stirring, 1 mL of methyl acetate was added for the washed NCs, or 1 mL of methyl acetate containing a desired amount of pyridine carboxamide was added for the *LE* NCs. Each mixture was stirred for an additional 1 min before being subjected to the same purification procedure as for the as‐synthesized NCs.

### 4.3 Device Fabrication

Indium tin oxide (ITO) substrate (200 nm) was cleaned sequentially by sonication in acetone and isopropanol for 15 min each. Cleaned substrates were exposed to ultraviolet‐ozone treatment for 20 min. *f*‐PEDOT:PSS composed of PEDOT:PSS (CLEVIOS P VP AI4083) and tetrafluoroethylene‐perfluoro‐3,6‐dioxa‐4‐methyl‐7‐octene‐sulfonic acid copolymer with a weight ratio of 2:1 were spin‐coated on ITO substrates. Then MHP NC solution was spin‐coated on HIL films as an EML. The LED devices were completed by sequential vacuum thermal evaporation of 2,2′,2″‐(1,3,5‐Benzinetriyl)‐tris(1‐phenyl‐1‐*H*‐benzimidazole) (TPBI, 50 nm), lithium fluoride (LiF, 1 nm) and Al (100 nm) under high vacuum (<5 × 10^−7 ^Torr). To fabricate HODs, the *f*‐PEDOT:PSS was spin‐coated onto the cleaned ITO substrates, followed by spin‐coating of the MHP NC solutions. Ag (100 nm) was subsequently thermally evaporated as the top electrode. To fabricate EODs, branched polyethylenimine (10 nm) was spin‐coated onto the cleaned ITO substrates. The MHP NC EMLs were spin‐coated and LiF and Al were then sequentially deposited following the same procedure used for LED devices fabrication.

### 4.4 Characterizations

pH measurements were obtained using a Checker Plus HI98100s‐pH tester. X‐ray photoelectron spectra were obtained using a Thermo Scientific Nexsa instrument with a monochromatic Al Kα line (1486.6 eV). Refractive indices *n*s of films were measured using a spectroscopic ellipsometer (Elli‐SE(UV)‐FM8, Ellipso Technology). Fourier transform infrared spectra were obtained using a Thermo‐Nicolet iS50 FTIR spectrometer. Zeta potentials were measured by Zetasizer Nano ZS90 analyzer. Transmission electron microscopy images were obtained using a JEOL‐JEM‐2100F. X‐ray diffraction patterns were recorded using a Rigaku miniFlex 600 diffractometer featuring a Cu *Kα* X‐ray source. Steady‐state photoluminescence (PL) and Time‐resolved PL (TCSPC FluoTime 300, PicoQuant) were conducted using a LDH‐P‐C‐405 (PicoQuant 405 nm pulse with 25 ps FWHM), a PDL 820 laser driver, and a UV‐red photomultiplier tube (PMT) monochromator (MSH300PQ‐0002, PicoQuant). UV‐vis absorption spectra were measured using Jasco V‐730 UV–visible Spectrophotometer. Fluorescence images were acquired using an Olympus BX51 microscope with a DP74 camera. The photoluminescence quantum yields were measured by using a Horiba Fluoromax‐4‐ with an integrating sphere at 405 nm excitation and 0.5 nm resolution. Water contact angles were measured using a contact‐angle goniometer (Phoenix‐MT(A), SEO): a 5 µL droplet of deionized water was gently dropped on the film surface, and the static contact angle was recorded at room temperature. Conductive atomic force microscopic measurements were performed using a Park XE7 (Park Systems) in contact mode, applying loading force of 1 nN to the tip; a bias of 0.1 V was applied to record the current between the tip and the sample. Ultraviolet photoelectron spectra were measured using a Thetaprobe (Thermo) equipped with a He I (21.2 eV) source, with a −10.0 eV bias applied to the sample. Capacitance–voltage *C*–*V* characteristics and electrochemical impedance spectroscopy (EIS) were measured using a Biologic SP‐300. *C‐* *V* data were acquired by sweeping the voltage from 0 to 10 V at a constant frequency of 1000 Hz. For EIS, measurements were conducted under dark conditions in the frequency range of 1 MHz to 100 mHz with an AC perturbation amplitude of 100 mV, and the bias was fixed at 0 V. Transient electroluminescence characteristics were measured using a pulse generator (hp 8116 A, 75 µs; 1 kHz frequency), PMT (PMTSS, Thorlabs) and oscilloscope (Agilent infiniium 54832D MSO). Current‐voltage‐luminance characteristics and luminous efficiencies of LEDs were measured using a source‐measurement unit (Keithley 2450), and a spectroradiometer (Komica‐Minolta CS‐2000), respectively.

### 4.5 Density Functional Theory

DFT calculations were performed using the Vienna Ab initio Simulation Package. The projector augmented‐wave method was used in conjunction with the generalized gradient approximation of Perdew–Burke–Ernzerhof for the exchange‐correlation functional. Grimme's DFT‐D3 method was used for van der Waals corrections. The plane‐wave basis set was expanded to a cutoff energy of 400 eV to minimize Pulay stress during the structural optimization. The structure was optimized by truncation until the Hellmann–Feynman forces were <0.03 eV Å^−1^. Electron‐energy convergence was set to 1 × 10^−4^ eV.

### 4.6 Optical Simulation

The light‐emitting behavior of MHP NC considering photon recycling were calculated using a method based on transfer matrix formalism [60, 61]. The device structure consists of glass (*n* = 1.50)/ITO (200 nm)/*f*‐PEDOT:PSS (*n* = 1.45, 70 nm) or PEDOT:PSS (*n* = 1.55, 70 nm)/MHP NC EML (30 nm)/TPBI (*n* = 1.75)/Al (100 nm). The *n* of ITO was adopted from literature [63], and the *n* of the MHP NC layer was assumed to be 1.90, while the extinction coefficient *k* was extracted from the experimentally measured absorption coefficient (Figure S51). The *n*s of *f*‐PEDOT:PSS (*n* = 1.45), PEDOT:PSS (*n* = 1.55), and TPBI (*n* = 1.75) were obtained via ellipsometry. The *n* of Al was obtained from literature [64]. The EML was modeled with 20 isotropic dipoles uniformly distributed over the MHP EML film, and the internal emission spectrum was assumed to be the same as the PL spectrum.

## Funding

This work was supported by the National Research Foundation of Korea (NRF) grant funded by the Korean government (Ministry of Science and ICT, MSIT) (RS‐2025‐00558546, RS‐2024‐00411892, RS‐2024‐00446129).

## Conflicts of Interest

The authors declare no conflicts of interest.

## Supporting information




**Supporting File**: adma73089‐sup‐0001‐SuppMat.pdf.

## Data Availability

The data that support the findings of this study are available from the corresponding author upon reasonable request.
