# Supplementary material for: Ligand‐Mediated Surface Carrier Modulation in Perovskite Nanocrystals for Charge‐Symmetric LEDs
Source: Adv Mater. 2026 Apr 19;38(28):e20499. doi: 10.1002/adma.202520499 (PMC13181538; doi:10.1002/adma.202520499)
Supplement: Supplementary file 1 — Supporting File: adma73089‐sup‐0001‐SuppMat.pdf. [file ADMA-38-e20499-s001.pdf]

Supporting Information

Ligand-Mediated Surface Carrier Modulation in Perovskite Nanocrystals for Charge-Symmetric LEDs

*Jongho Park[1,‡], Jisu Ha[1,‡], Daehwan Kim[1,‡], Hyeon Woo Kim[2,3], Han Uk Lee[4], Boseung Je[5], Dodam Kim[1], Jun-Su Yeo[1], Min Gyo Kim[1], Changsoon Cho[5,6], Sung Beom Cho[2,4], and Tae-Hee Han[1,7]**

[1]Division of Materials Science and Engineering, Hanyang University, Seoul, Republic of Korea.

[2]Department of Materials Science and Engineering, Ajou University, Suwon, Republic of Korea.

[3]Department of Mechanical Engineering & Material Science, Washington University in St. Louis, St. Louis, Missouri, United States.

[4]Department of Energy Systems Research, Ajou University, Suwon, Republic of Korea.

[5]Department of Materials Science and Engineering, Pohang University of Science and Technology (POSTECH), Pohang, Republic of Korea.

[6]Institute for Convergence Research and Education in Advanced Technology, Yonsei University, Seoul, Republic of Korea.

[7]Department of Display Science and Engineering, Hanyang University, Seoul, Republic of Korea.

Supporting Notes

Supporting Note 1

According to density functional theory (DFT) calculations, due to the larger ionic radius of GA^+ compared to FA^+ and the relatively higher formation energy of $GAPbBr_3$ (0.34 eV) compared to that of $FAPbBr_3$ (0.20 eV), substitutional doping of GA into the FA-based MHP NCs is only possible up to low concentrations (Figure S1).

Considering the energy evolution of $FA_{1-x}GA_xPbBr_3$ according to GA^+ doping concentration x, there exists an optimal substitutional doping concentration around $x \approx 0.1$.[1] This limit prevents GA^+ from persisting within the lattice, so excess GA^+s locate on the surface of MHP NCs when GA^+ is higher than the limit. Segregation energy E_{SEG} was defined as the difference in total energies between the systems with the GA located at the surface and in the bulk lattice. A negative E_{SEG} indicates that the segregation of GA toward the surface is favored in the system. The E_{SEG} with GA on the surface was calculated to be negative for both FABr (100) termination (E_{SEG_FABr} = -0.54 eV) and $PbBr_2$ (100) termination (E_{SEG_PbBr2} = -0.25 eV) cases (Figures S2 and S3).

The theoretical predictions regarding GA^+ doping were experimentally validated using X-ray diffraction (XRD) analysis of the (100) plane in $FAPbBr_3$ (Figure S4). Upon substitutional incorporation of GA^+, the larger GA^+ expands the lattice, thereby shifting the (100) diffraction peak to lower 2θ (larger d-spacing) and decreasing from 14.86° in $FAPbBr_3$ to 14.79° in $FA_{0.9}GA_{0.1}PbBr_3$, consistent with substitutional incorporation of an oversized A-site cation. However, as the GA fraction increases beyond ~ 10%, the (100) peak position becomes nearly unchanged, indicating that the bulk lattice parameter no longer expands even though the nominal GA content increases, because the excess GA^+ segregated to the NC surface. The result is a $FA_{0.9}GA_{0.1}PbBr_3$ (t = 1.0135) core surrounded by a GA^+-rich shell, forming a chemically induced core-shell-like configuration in $FA_{0.816}GA_{0.144}Cs_{0.04}PbBr_3$ NCs (Figure S5).

Supporting Note 2

The surface X/Pb ratio of metal halide perovskite nanocrystals (MHP NCs) typically deviates from the bulk stoichiometric value of approximately 3. When the surface X/Pb ratio exceeds 3, the MHP NCs tend to adopt an ($APbX_3$) (PbX_2) (AX)-type structure with an AX-termination. Conversely, when the surface X/Pb ratio falls below 3, an ($APbX_3$) (AX) (PbX_2)-type structure with a PbX_2-termination is favored. According to X-ray photoelectron spectroscopy (XPS) analysis, the Br/Pb ratios of the as-synthesized, washed, and ligand-exchanged (*LE*) NCs are 2.28, 2.23, and 2.52, respectively, suggesting that the MHP NC surfaces are predominantly terminated with $PbBr_2$ (Figure S6).[2]

In the synthesized MHP NCs, a molar ratio of approximately 8:1 between oleic acid (OA) and decylamine (DAm) was employed, resulting in OA predominantly occupying the surface ligands. Through an acid-base reaction, OA and DAm are converted into oleate$^-$ and decylammonium$^+$ species, respectively.[3] Consequently, oleate$^-$ preferentially coordinates with surface Pb^{2+} ions, while decylammonium$^+$ interacts electrostatically with Br^- at the MHP NC surface, consistent with the $PbBr_2$-termination.

Supporting Note 3

In the ^1H NMR spectrum of OA, the characteristic carboxylic acid (COOH) proton appears at ~12.0 ppm, while the alkene protons are at ~5.3 ppm. Based on the molecular structure of OA, the expected proton integration ratio between carboxylic acid and alkene protons is 1:2, which is consistently observed in the reference spectrum (Figures S18 and S19).

In contrast, the spectrum of the as-synthesized NCs showed a markedly different integration behavior. When the carboxylic acid proton is used as a reference, the integration ratio between carboxylic acid and alkene protons becomes approximately 1:8. This deviation indicates that a significant fraction of OA is present in the oleate form bound to the NC surface, consistent with the description in the manuscript (Figures S18 and S19).

Upon ligand washing using MeOAc, the integration ratio between carboxylic acid and alkene protons recovers to 1:2, identical to that of OA. This result indicates that MeOAc effectively removes surface-bound oleate species attributed to protonation of oleate by H^+ generated during hydrolysis of MeOAc (Figure S20), leading to complete desorption of oleate ligands from the NC surface. This observation directly demonstrates the effectiveness of hydrolysis-based washing process described in the manuscript (Figure S18-S20).

Importantly, after PCA ligand exchange, the carboxylic acid-to-alkene proton ratio remains unchanged at 1:2, demonstrating that neutral PCA does not induce ligand stripping or further desorption of oleic acid/oleate species. Instead, the successful adsorption of PCA is evidenced by the emergence of distinct PCA proton signals in the 7-9 ppm region. These PCA-related peaks remain clearly observable even after multiple purification steps, confirming that PCA is stably adsorbed on the NC surface (Figures S18 and S19).

Supporting Note 4

To estimate the surface energy γ_s of the NC films, the water contact angles were analyzed using a simplified empirical model based on Neumann's equation, which relates the contact angle to γ as: $\gamma_s = \gamma_l(\frac{1+cos\theta}{2})^2$ where γ_l is the surface tension of water (~72.8 mN/m at 20°C).[4] Based on this model, the surface energies of the as-synthesized NCs (contact angle ≈ 8.6°) and the washed NCs (contact angle ≈ 9.2°) are estimated to be ~72.0 mN/m and ~71.9 mN/m, respectively, whereas that of the *LE* NCs (contact angle ≈ 27.1°) is reduced to ~65.0 mN/m (Figure S26). This decrease in surface energy indicates a clear transition toward a more hydrophobic surface in the *LE* NCs, consistent with the introduction of PCA that forms a compact and well-organized surface layer. In contrast, the washed NCs, capped with short and polar acetate ligands, retain high surface energy and strong hydrophilicity.

Supporting Note 5

To directly investigate whether ligand exchange enhances electronic coupling among NCs, we experimentally determined the exciton diffusion length (L_D) of the NC films before and after ligand exchange using a well-established exciton-quenching model commonly employed in OLEDs.[5,6]

We prepared MHP NC films with thicknesses ranging from 15 to 120 nm on two different substrates: (i) quartz (non-quenching) and (ii) PEDOT:PSS-coated quartz (assuming PEDOT:PSS as nearly perfect quenching layer).[6]

Since PEDOT:PSS acts as an exciton sink, the PL intensity difference between the two substrates reflects the extent of exciton diffusion toward the quenching interface. Because we assumed that excitons reaching the PEDOT:PSS interface are efficiently quenched, the difference in PL intensity, defined as $PL_{quartz} - PL_{PEDOT:PSS}$, directly reflects the fraction of excitons that diffuse to the quenching interface, and thus provides insight into the extent of exciton diffusion.

The experimental results were fitted to a theoretical model with the steady-state 1D exciton diffusion under continuous generation (Equation S1):

$$0 = L_D \frac{\partial^2 p(x)}{\partial x^2} - p(x) + \tau_D G \quad \text{(S1)}$$

where L_D is the exciton diffusion length, x is the film thickness of the EML, p is exciton density, τ is the exciton lifetime, and G is the exciton generation rate.[5,6]

Boundary conditions are used: (i) the interfaces at the EML/air and inert substrate/EML are ideally non-quenching interfaces ($p(x = 0) = 0$), (ii) the interface between the EML and a quenching material film is assumed to have perfectly fast-quenching interface ($\left.\frac{\partial p}{\partial x}\right|_{x=d} = 0$), (iii) exciton generation rate G_0 is uniform throughout the thin film ($G(x) = G_0$).

By using the above boundary conditions, Equation S1 can be solved into fraction of excitons reaching the quenching interface:

$$\eta = \frac{L_D}{d} \frac{\left[1 - \exp\left(-\frac{2d}{L_D}\right)\right]}{\left[1 + \exp\left(-\frac{2d}{L_D}\right)\right]} \quad \text{(S2)}$$

Because PL intensity is proportional to the average exciton density, the PL intensities of films with ($PL_{PEDOT:PSS}$) and without (PL_{quartz}) quencher can be used for determination of exciton diffusion length (L_D) of the film. Hence, by measuring the PL intensities as a function of film thickness, ɲ= 1 − $PL_{PEDOT:PSS}$ / PL_{quartz} can be measured. Consequently, L_D is determined by fitting $PL_{PEDOT:PSS}$ /PL_{quartz} to Equation S3.

$$\frac{PL_{PEDOT:PSS}}{PL_{quartz}} = 1 - \frac{L_D}{d}\frac{\left[1-\exp\left(-\frac{2d}{L_D}\right)\right]}{\left[1+\exp\left(-\frac{2d}{L_D}\right)\right]} \quad \text{(S3)}$$

As shown in Figure S27, the *LE* NC films exhibit a large and nearly thickness-independent PL intensity difference ($PL_{quartz}-PL_{PEDOT:PSS}$), which suggests that a significant portion of excitons reaches the quenching interface even at large thicknesses. In contrast, the as-synthesized NC films showed a strong thickness dependence; as the film becomes thicker, the PL difference rapidly decreases, indicating that fewer excitons can diffuse through the bulk and reach the quenching layer.

This trend clearly demonstrates that exciton diffusion is substantially enhanced in *LE* NC films. By fitting the normalized PL ratio $PL_{PEDOT:PSS}/PL_{quartz}$ vs. film thickness according to the theoretical model based on steady-state 1D exciton diffusion (Equation S3 and Figure 3h), we extracted the exciton diffusion lengths: L_D = 20.42 ± 0.67 nm for the as-synthesized NCs, and L_D = 69.23 ± 2.85 nm for the *LE* NCs.

Supporting Note 6

Upon ligand exchange with PCA, the MHP NC surface undergoes a significant alteration in its interfacial electronic environment. The introduction of PCA leads to the formation of molecular dipoles at the MHP NC surfaces, which originate from the inherent asymmetry in the electronic structure of the PCA. Specifically, the electron-rich pyridine ring of PCA preferentially adopts an orientation pointing away from the MHP NC surface, toward the vacuum interface. This molecular alignment results in a well-defined surface dipole, in which the negative pole (associated with the pyridine ring) is directed outward (Figure S30).

The formation of such surface dipoles directly influences on the energy levels of the MHP NCs. Outward-facing dipoles with negative termini induce an electrostatic potential that shifts the vacuum level upward, thereby increasing the material's work function (Figure S30).[7]

This proposed mechanism is further substantiated by zeta-potential measurements (Figure 2i,j), which reveal a noticeable negative shift following PCA treatment. This shift indicates a more negatively-charged surface environment, consistent with the exposure of the electron-dense pyridine ring at the outermost interface.

Supporting Note 7

Transient electroluminescence (TREL) measurements were conducted to identify the dominant majority carrier in our MHP NC-based LED system by comparing devices with different hole injection properties. The device using *f*-PEDOT:PSS, which facilitates enhanced hole injection, exhibited a significantly shorter rise time (~1.60 ms) than the PEDOT:PSS-based device (~6.72 ms) (Figure S32). Despite the longer hole transport distance in the *f*-PEDOT:PSS-based device, the faster rise time indicates that holes are the dominant majority carriers. These results also suggest that the recombination zone is located near the interface between the emissive layer and the electron transport layer, where electrons encounter accumulated holes.[8–12]

Supporting Note 8

Acidic ligands (hexylphosphonic acid (HPA), benzylphosphonic acid (BPA)) severely degraded the photophysical properties of the NCs, reducing both PL intensity and exciton lifetime due to ligand stripping and the generation of undercoordinated traps (Figure S34 and Table S3).[13] Both the PL intensity and the τ_{avg} decreased sharply, with τ_{avg} reduced to approximately 7.4 ns for HPA and 4.7 ns for BPA from 10.3 ns (as-synthesized), indicating severe exciton quenching originating from disruption of the surface acid-base equilibrium, whereby acidic ligands strip pre-existing surface ligands and generate undercoordinated surface sites (Figure S34b,c and Table S3).[13] As a consequence of this ligand-stripping process, the colloidal stability was severely compromised for HPA-treated NCs: while PCA-treated NCs retained approximately 90% of their initial PL intensity even after 450 h, the PL intensity of HPA-treated NCs rapidly decreased to ~70% within ~100 h, indicating accelerated optical degradation (Figure S35).

These NCs also exhibited poor LED characteristics, with increased interfacial charge accumulation and inefficient recombination, as evidenced by C-V measurements (Figure S36) and low EQEs (Figure S37).

The neutral benzamide (BZA) preserved the optical properties of the as-synthesized NCs, with both PL intensity and τ_{avg} remaining comparable (11.7 ns) to as-synthesized NCs (10.3 ns), suggesting that neutral coordination avoids acid-induced nonradiative pathways but provides only limited surface passivation (Figure S34b,c and Table S3).

In addition, devices treated with the neutral BZA showed a reduced maximum capacitance, suggesting partial mitigation of charge accumulation. However, the capacitance decreased only gradually at higher voltage, resulting in a noticeably flatter slope compared to that with PCA (Figure S36). This is attributed to its inert benzene ring, which lacks specific orbital interactions with surface metal centers. Although it avoids acid-induced trap formation, BZA does not actively contribute to electronic coupling or surface dipole modulation.

For devices employing ligands other than PCA (i.e., HPA, BPA, and BZA), both the current density and luminance decreased compared to the as-synthesized devices (Figure S37). These results indicate that either acidic ligands or neutral benzene (non-hetero aromatic) ligands cannot support efficient charge recombination in operating devices.

Supporting Note 9

We designed three representative compositions: single-cation (FA, $FAPbBr_3$), binary (FA-GA, $FA_{0.815}GA_{0.185}PbBr_3$), and ternary (FA-GA-Cs, $FA_{0.816}GA_{0.144}Cs_{0.04}PbBr_3$) for this comparison. These were synthesized and fabricated into LEDs under identical conditions, including the same PCA ligand-exchange treatment.

Photophysically, PLQY increased stepwise from 71.3% (FA) → 78.0% (FA-GA) → 88.2% (FA-GA-Cs) (Figure S38), and this trend correlates with a steady reduction in hole trap density (n_t), as extracted from hole-only devices (Figure S39). These results confirm that A-site alloying effectively suppresses defect states and improves radiative efficiency.

To quantitatively assess defect-related losses in the NC films, hole-only devices with an ITO/*f*-PEDOT:PSS/MHP NC EML/Ag structure were fabricated to extract the hole trap density n_t in the NC EML (Figure S39a). From the *J-V* curves, the trap-filled limit voltage V_{TFL} was determined, and n_t was calculated using,

$$n_t = \frac{2V_{TFL}\varepsilon_0\varepsilon_r}{eL^2} \quad \text{(S4)}$$

where ε_0 is the vacuum permittivity, ε_r is the relative dielectric constant (43.6 for $FAPbBr_3$),[14] e is the elementary charge and L is the thickness of the MHP NC layer (Figure S39b,c). The extracted hole trap densities are 6.20×10^{17} cm^{-3} for the single-cation (FA) NC device, 3.71×10^{17} cm^{-3} for binary-cations (FA-GA) NC device, and 3.14×10^{17} cm^{-3} for ternary-cations (FA-GA-Cs) NC devices (Figure S39c). This reduction in n_t indicates that A-site engineering suppresses trap states that otherwise promote space-charge accumulation and trap-assisted nonradiative recombination in LEDs.

To evaluate how these translate into actual LED device performance, we fabricated LEDs with each composition using an identical device architecture. The luminance and luminous efficiency characteristics showed clear improvement from single to ternary A-site compositions (Figures S40 and S41). The ternary FA-GA-Cs NC LEDs exhibited higher luminance at a given voltage, increased current and external quantum efficiencies, and suppressed efficiency roll-off, indicating better charge balance and recombination.

Supporting Note 10

For inorganic $CsPbBr_3$ NCs, the *LE* NCs exhibited a distinct peak at 1,710 cm^{-1}, which corresponds to the C=O stretching vibration in FTIR spectrum (Figures S42a and S43a). This peak originates from the carbonyl group of PCA and appears at 1,698 cm^{-1} in free PCA.[15] The observed blueshift indicates strong coordination of the C=O carbonyl oxygen to surface Pb^{2+} of MHP NCs; this process increases both C=O bond order and vibrational frequency.[16] Additional PCA-specific peaks appeared in the 1,650-1,550 cm^{-1} region (N-H bending and C=N stretching) (Figures S42a and S43a), and a sharp C-N stretching peak at 1,200 cm^{-1} intensified with increasing PCA concentration (Figures S42b and S43b).[17,18] Importantly, these FTIR features and their evolution closely match those observed for ternary A-site MHP NCs ($FA_{0.816}GA_{0.144}Cs_{0.04}PbBr_3$), confirming that PCA ligands are effectively coordinated to $CsPbBr_3$ NCs in a manner fully consistent with the mixed-cation system.

^{1}H NMR spectra of $CsPbBr_3$ NCs were measured to further verify the ligand exchange with PCA. Characteristic resonances attributed to PCA were clearly observed in 7-9 ppm region, confirming the presence of PCA on the $CsPbBr_3$ NC surface (Figure S44). For the synthesis of $CsPbBr_3$ NCs, didodecyldimethylammonium (DDAB) was employed as the native ligand. By integrating the PCA signals relative to the NH_2^+-associated proton signals of DDAB as an internal reference, the ligand composition of the final *LE* $CsPbBr_3$ NCs was quantitatively estimated. This analysis revealed that the surface ligand ratio in the *LE* $CsPbBr_3$ NCs was approximately DDAB:PCA = 3:1, indicating effective partial ligand exchange with PCA.

For $FAPbBr_3$ and $MAPbBr_3$ NCs, the steady-state PL intensity increased significantly (Figures S45a, and S46a), and TRPL analysis revealed prolonged carrier lifetimes, consistent with effective surface passivation (Figures S45b, S46b and Tables S4, S5). *C-V* measurements confirmed more balanced electron-hole injection and transport in the *LE* NC devices, as indicated by a reduced peak capacitance at lower voltage and a steeper drop at high bias (Figure S47). Correspondingly, the device exhibited a ~4-fold increase in maximum luminance and a substantial improvement in current efficiency and EQE improvement from 14.3% (as-synthesized $FAPbBr_3$ NCs) to 23.1% (*LE* $FAPbBr_3$ NCs) (Figure S48).

Supporting Note 11

We measured the operational half-lifetime LT_{50}, defined as the time required for the luminance to decrease to 50% of its initial value, under constant current operation. The as-synthesized NC device exhibited an LT_{50} of approximately 1.2 h at an initial luminance of 100 cd m^{-2}, whereas the *LE* NC device showed an improved LT_{50} of ~5.0 h at an elevated initial luminance of 170 cd m^{-2} (Figure S50). Using the commonly adopted acceleration model ($LT \propto L^{-n}$, where $n = 1.84$),[19] this corresponds to an estimated LT_{50} of ~13.0 h at an initial luminance of 100 cd m^{-2}, demonstrating a more than 10-fold improvement in operational stability when compared under equivalent luminance conditions.

Supporting Figures

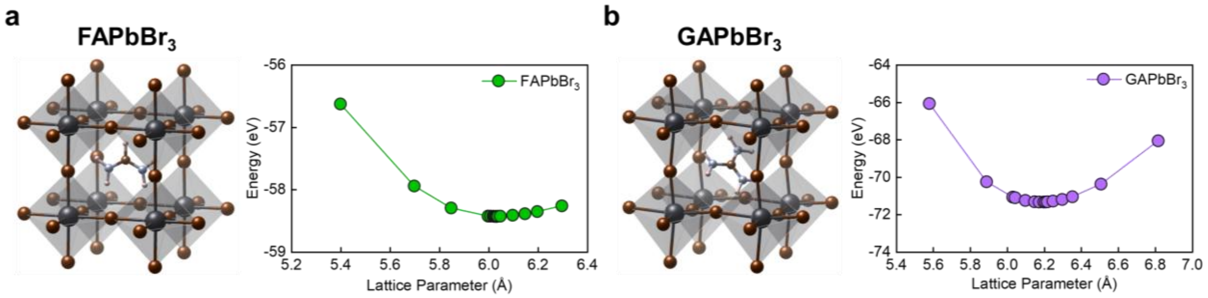

Figure S1. The optimized structures of unit cell and lattice parameter for a) $FAPbBr_3$, and b) $GAPbBr_3$.

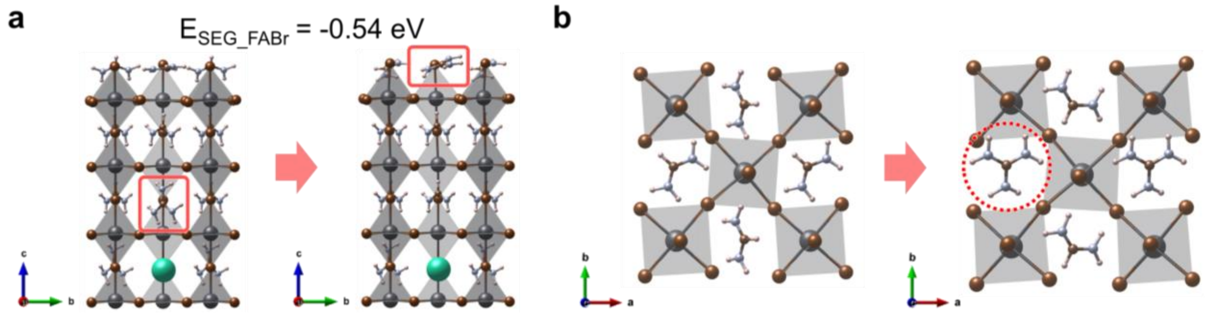

Figure S2. a) Side view and b) top view of calculated structures of perovskite surface with FABr-termination when GA is inside the bulk (left) and when GA is exposed on the surface (right).

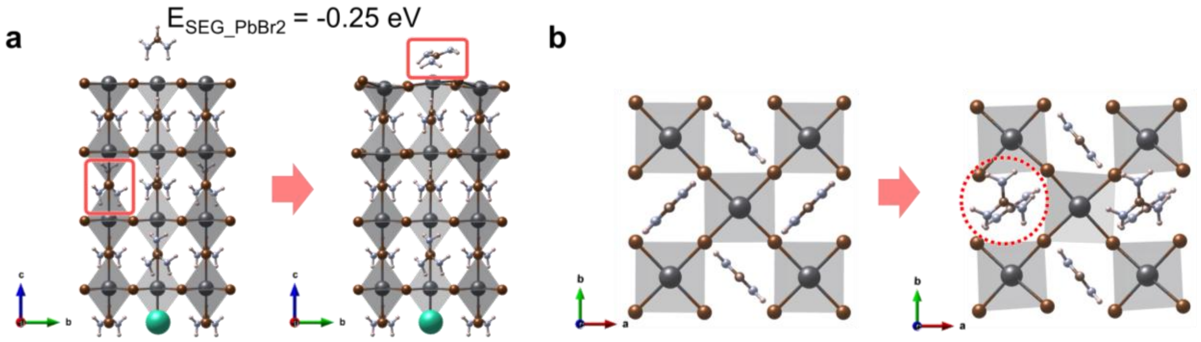

Figure S3. a) Side view and b) top view of calculated structures of perovskite surface with $PbBr_2$-termination when GA is inside the bulk (left) and when GA is exposed on the surface (right).

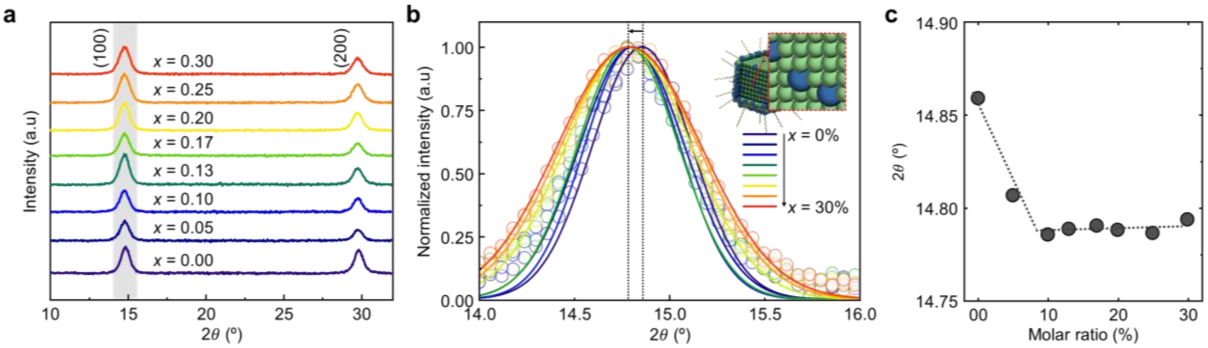

Figure S4. a) XRD patterns of $FA_{1-x}GA_xPbBr_3$ MHP NC films with varying GA content x and b) evolution of 2θ extracted from (100) of $FA_{1-x}GA_xPbBr_3$ MHP NC films.

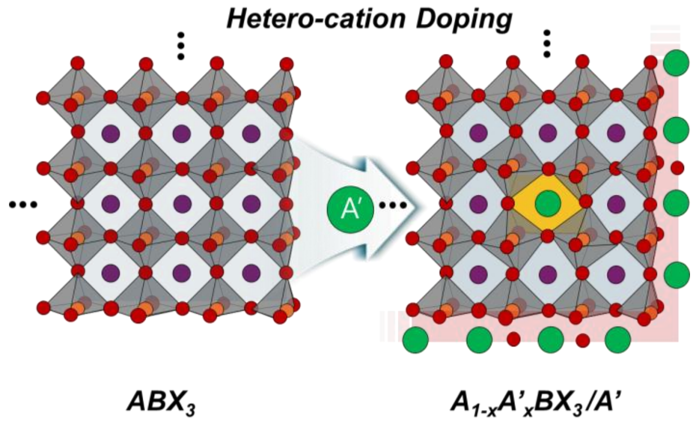

Figure S5. Schematic illustration of excess GA^+ surface segregation forming an FA-rich core and a GA^+-rich shell.

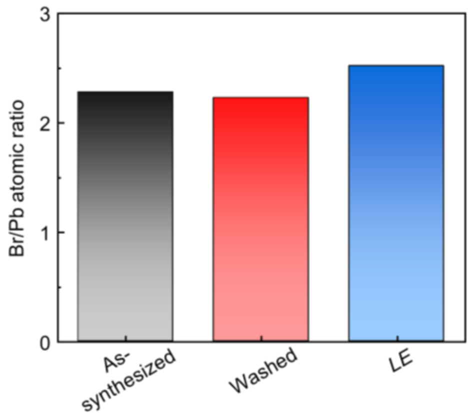

Figure S6. Evolution of Br to Pb ratio of the as-synthesized, washed, and *LE* NCs.

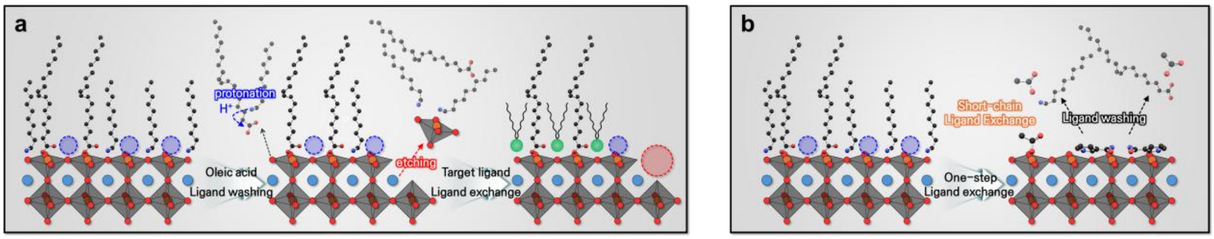

Figure S7. Schematic illustrations of the ligand-exchange mechanism for MHP NCs and their resulting effects using a) OA, and b) both MeOAc and PCA.

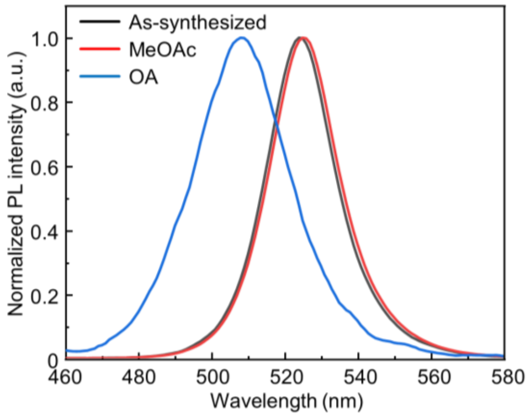

Figure S8. Normalized PL intensities of colloidal NC solutions without treatment (as-synthesized), and treated with MeOAc or OA.

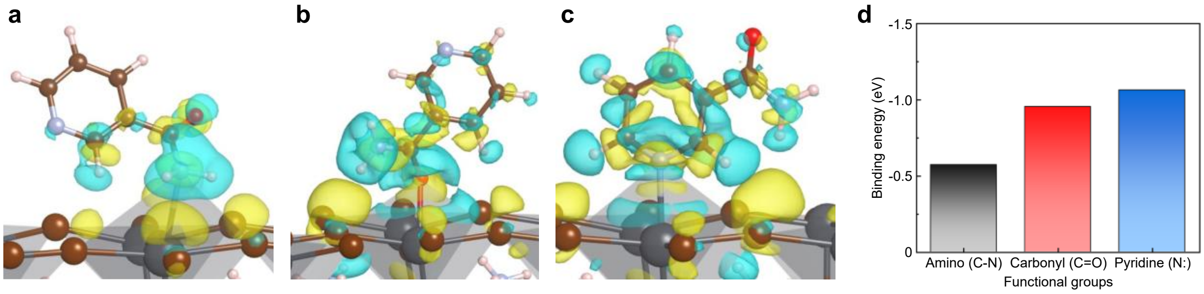

Figure S9. Most favorable molecular configurations of PCA coordinated individually to the undercoordinated Pb^{2+} sites via the a) amino group, b) the carbonyl group, and c) the pyridine groups. d) The binding energies of PCA with different functional groups (amino, carbonyl, and pyridine) coordinated to the undercoordinated Pb^{2+} sites.

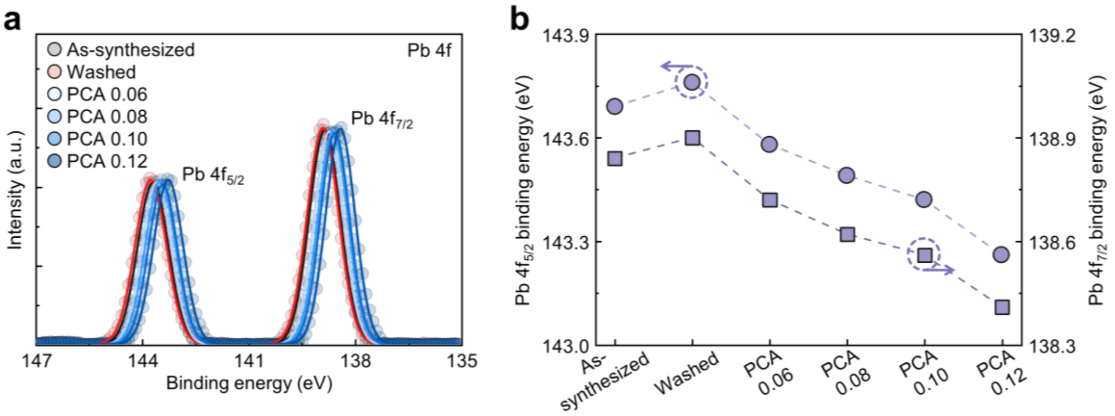

Figure S10. a) XPS spectra of Pb 4f for the as-synthesized, washed and *LE* NCs with varying PCA concentrations. b) Binding energy shifts of Pb 4f peaks for the as-synthesized, washed, and *LE* NCs with increasing PCA concentrations, extracted from the fitted XPS spectra.

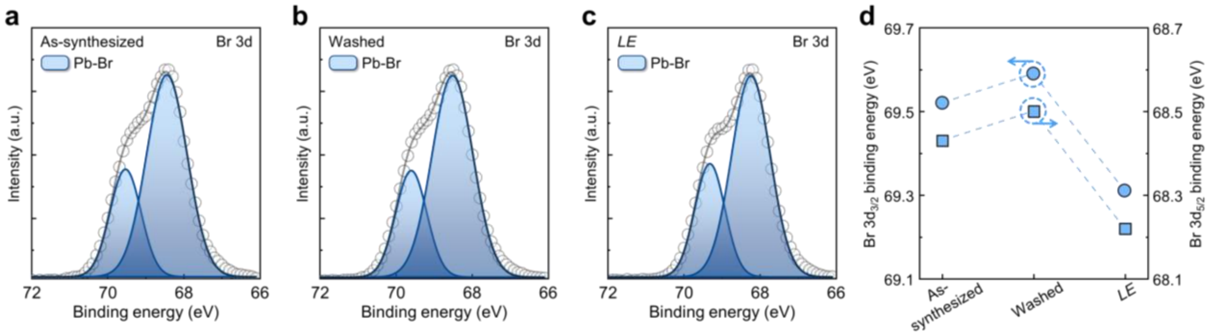

Figure S11. XPS spectra of Br 3d for the a) as-synthesized, b) washed, and c) *LE* NCs. d) Binding energy shifts of Br 3d peaks for the as-synthesized, washed, and *LE* NCs extracted from the fitted XPS spectra.

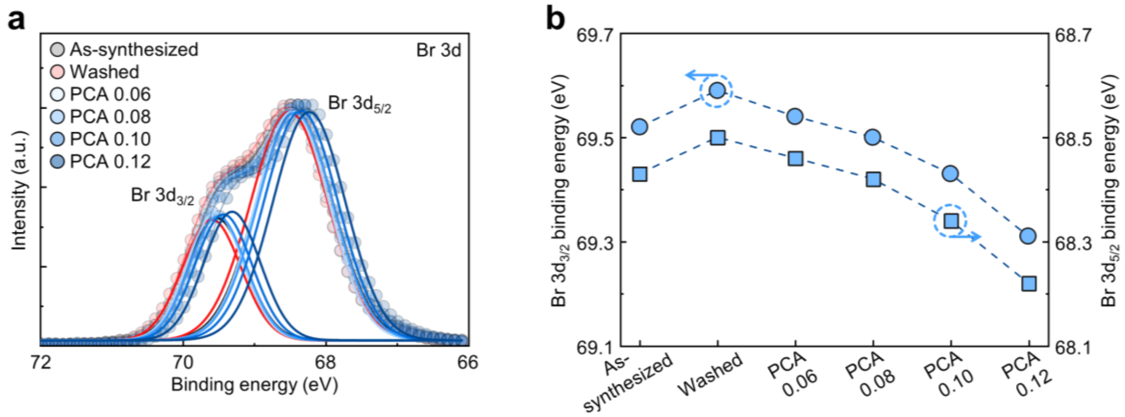

Figure S12. a) XPS spectra of Br 3d for the as-synthesized, washed and *LE* NCs with varying PCA concentrations. b) Binding energy shifts of Br 3d peaks for the as-synthesized, washed, and *LE* NCs with increasing PCA concentrations, extracted from the fitted XPS spectra.

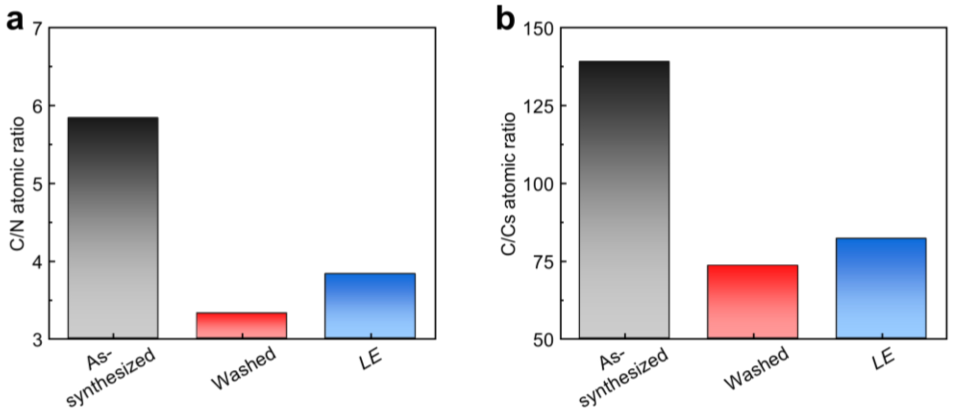

Figure S13. Evolution of a) C to N ratio**,** and b) C to Cs ratio of the as-synthesized, washed, and *LE* NCs.

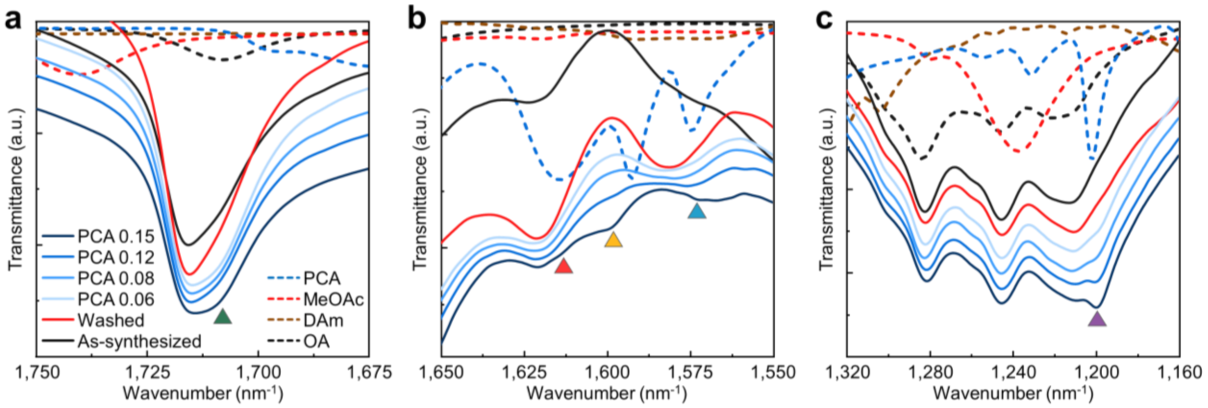

Figure S14. FTIR spectra of the ligands (OA, DAm, MeOAc, and PCA) and MHP NCs with varying PCA concentrations for a) C=O stretching, b) N-H bending and C=N stretching, and c) C-N stretching.

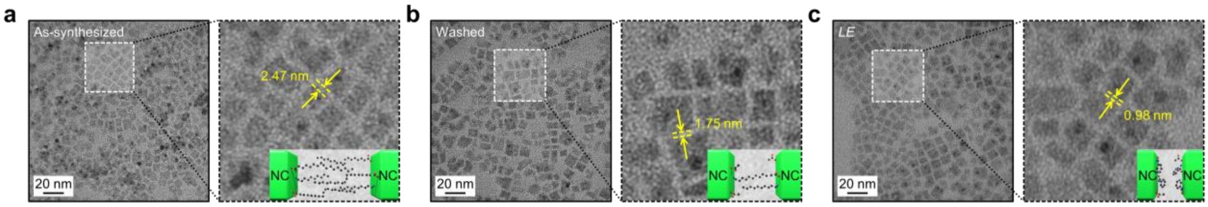

Figure S15. TEM images and inter-crystal distance of the a) as-synthesized, b) washed, and c) *LE* NCs.

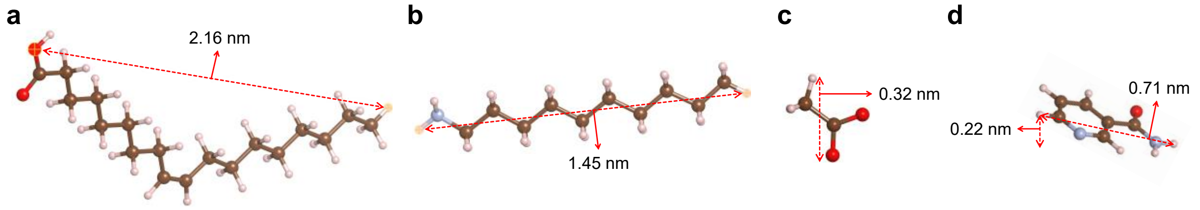

Figure S16. Molecular lengths of the ligands, a) OA, b) DAm, c) Ac^-, and d) PCA, calculated from optimized structures.

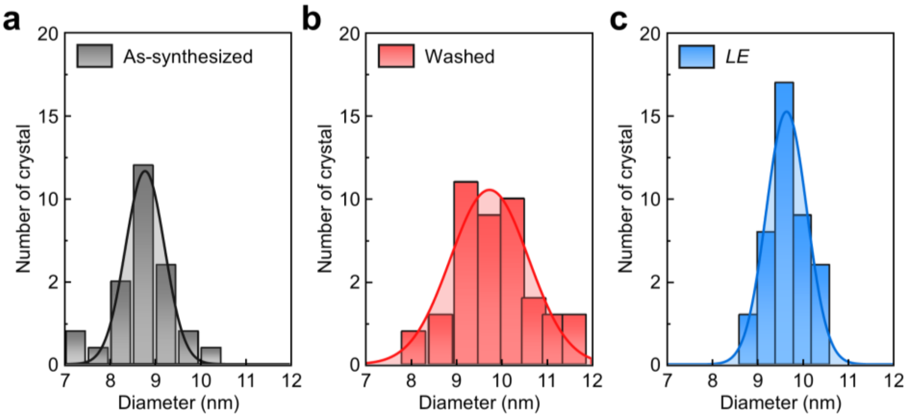

Figure S17. Statistical distributions of the a) as-synthesized, b) washed, and c) *LE* NC diameters extracted from the TEM images.

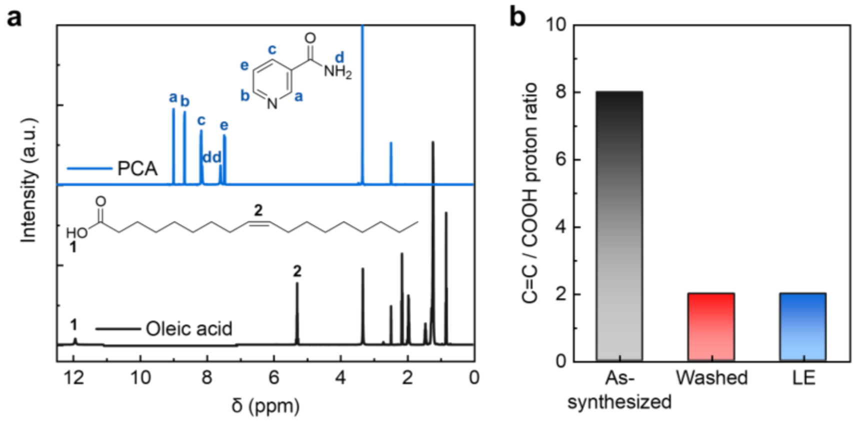

Figure S18 a) Molecular structures of PCA (left) and OA (right), and b) ^{1}H NMR spectra of the as-synthesized, washed and *LE* NCs, and c) corresponding C=C / COOH proton ratios extracted from the integration of the ^{1}H NMR spectra.

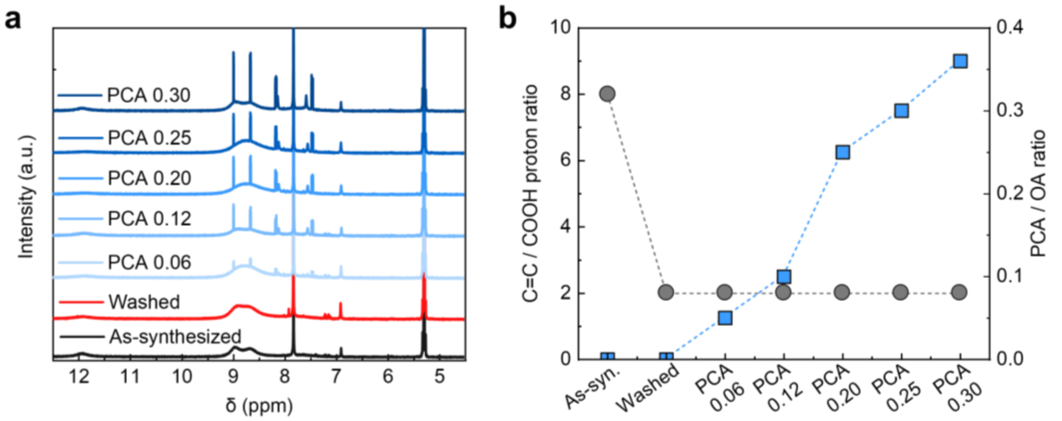

Figure S19. a) ^{1}H NMR spectra of the as-synthesized, washed and *LE* NCs with varying PCA concentrations, and b) corresponding C=C / COOH proton ratios (black) and PCA / OA ligand ratios (blue) extracted from the integration of the ^{1}H NMR spectra.

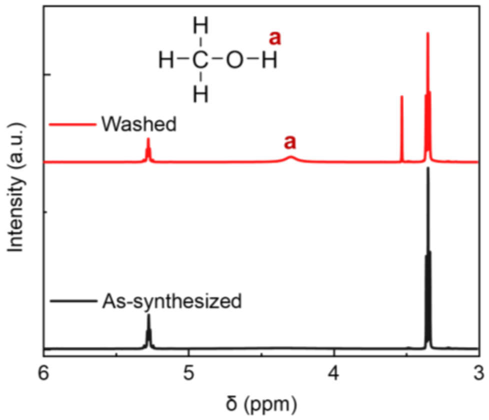

Figure S20. ^{1}H NMR spectra of the as-synthesized and washed NCs before purification process.

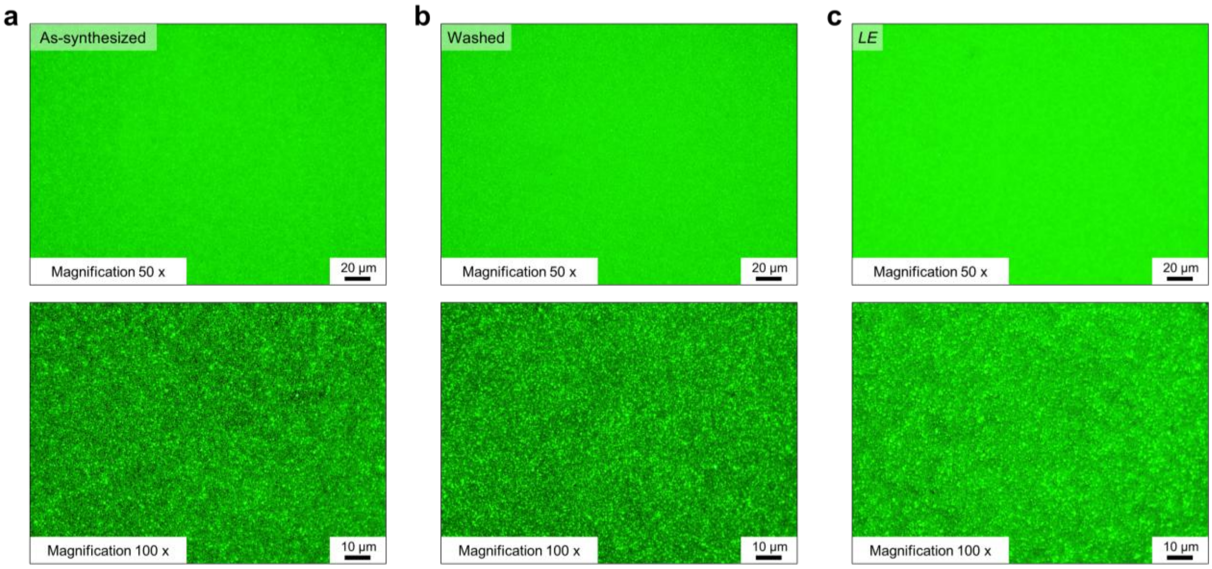

Figure S21. Fluorescence microscopy images of the a) as-synthesized, b) washed, and c) *LE* NCs.

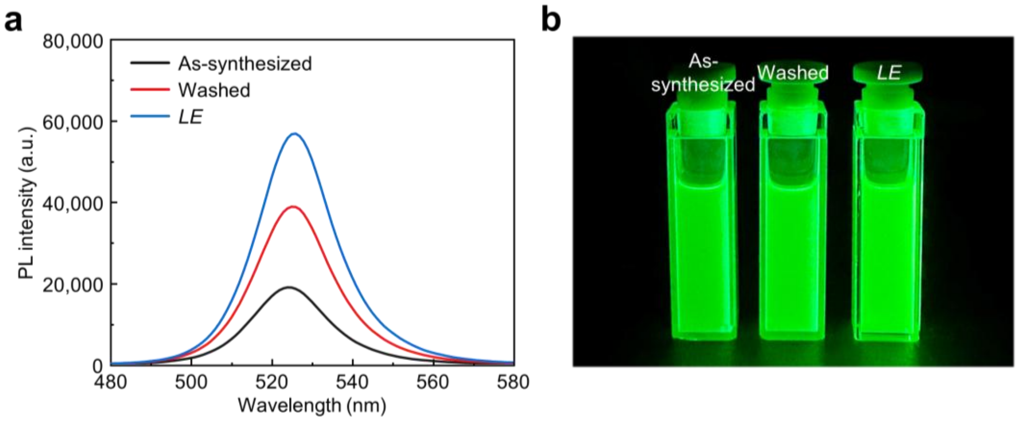

Figure S22. a) UV-vis absorption and steady-state PL spectra. b) Optical image of the colloidal MHP NC solutions under 365 nm UV illumination.

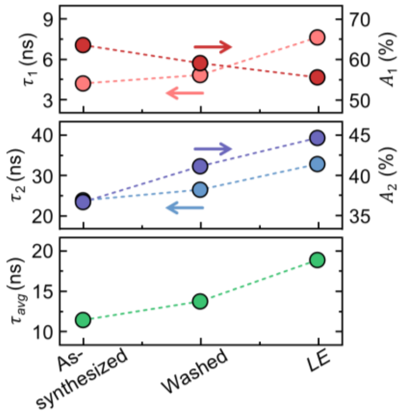

Figure S23. PL lifetimes (τ_1, τ_2, and τ_{avg}) extracted from TRPL decay curves of MHP NC films.

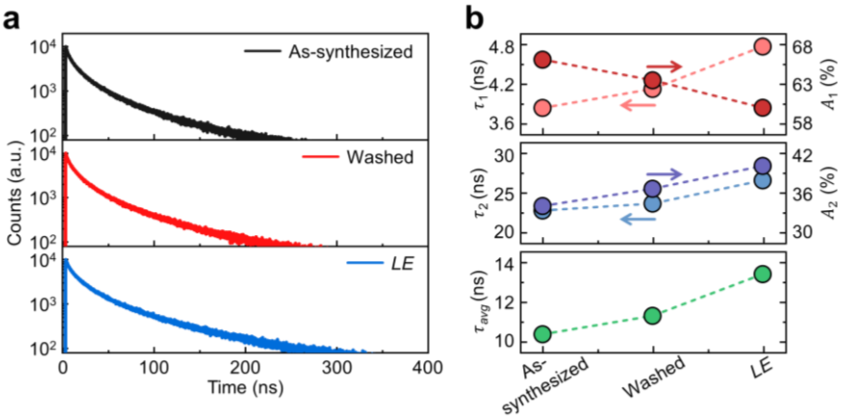

Figure S24. a) TRPL of colloidal MHP NC solutions. b) PL lifetimes (τ_1, τ_2, and τ_{avg}) extracted from TRPL decay curves.

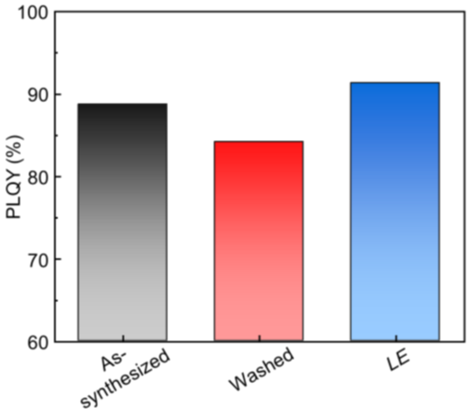

Figure S25 PLQYs of the as-synthesized, washed, and *LE* NC solutions.

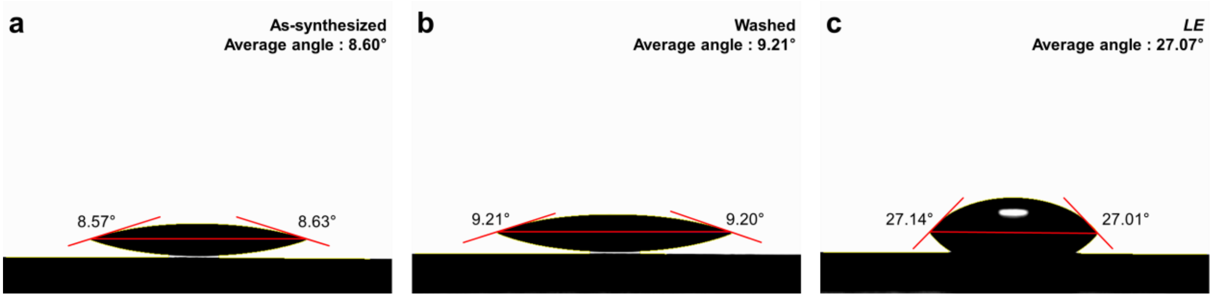

Figure S26 Contact angle of water droplet on the a) as-synthesized, b) washed, and c) *LE* NC surface.

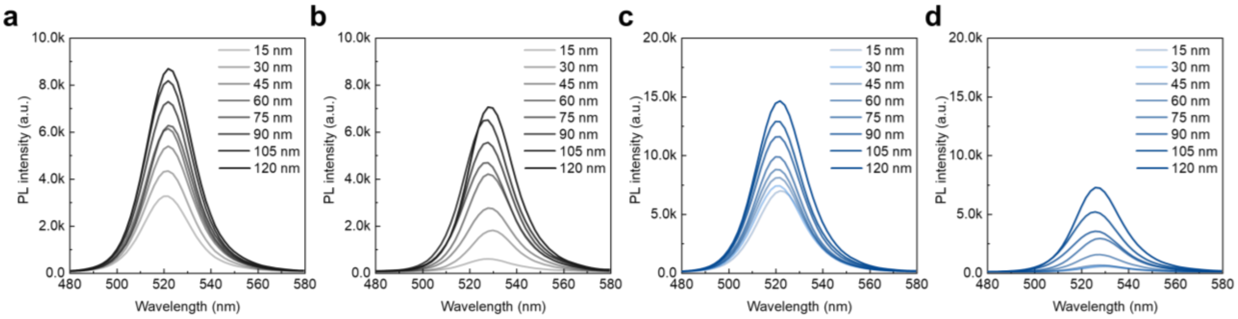

Figure S27. PL intensities of the as-synthesized NC films deposited on a) quartz substrates, and b) PEDOT:PSS, and the *LE* NC films deposited on c) quartz substrates, and b) PEDOT:PSS in varying EML NC thickness.

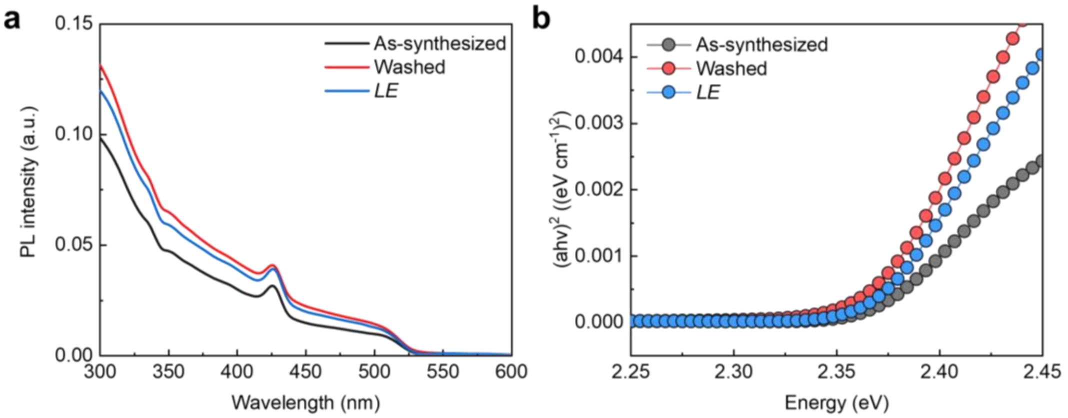

Figure S28. a) UV-vis absorption, and b) tauc plot of the as-synthesized, washed, and *LE* NC films.

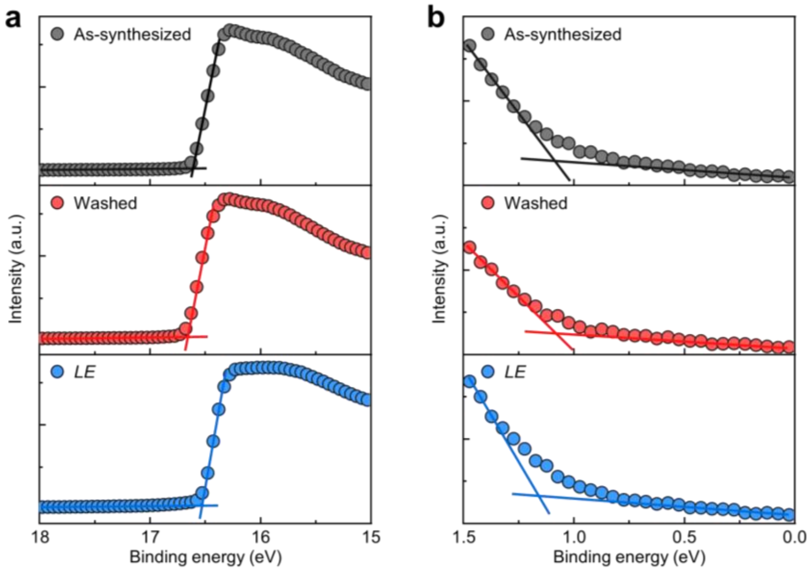

Figure S29. UPS spectra of MHP NC films showing a) the secondary electron cut-off**,** and b) the valence band onset.

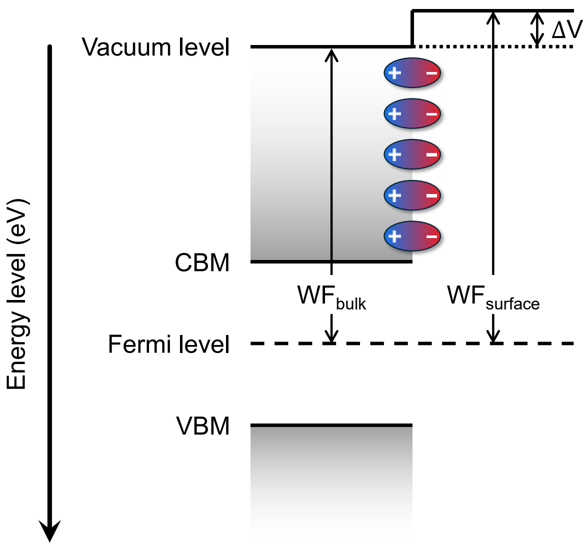

Figure S30. Schematic illustration of how PCA-induced surface dipoles affect the perovskite energy band diagram.

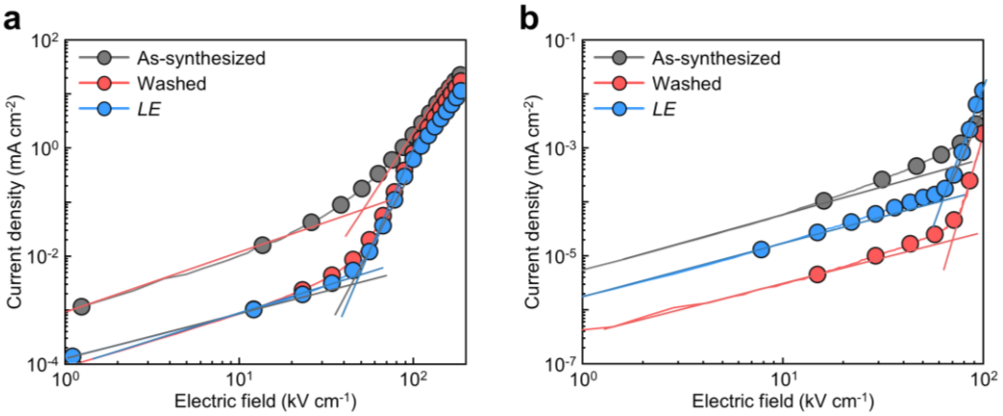

Figure S31. Current density-electric field characteristics of a) hole-only and b) electron-only devices, showing the trap-filled limit regime for the as-synthesized, washed, and *LE* NCs.

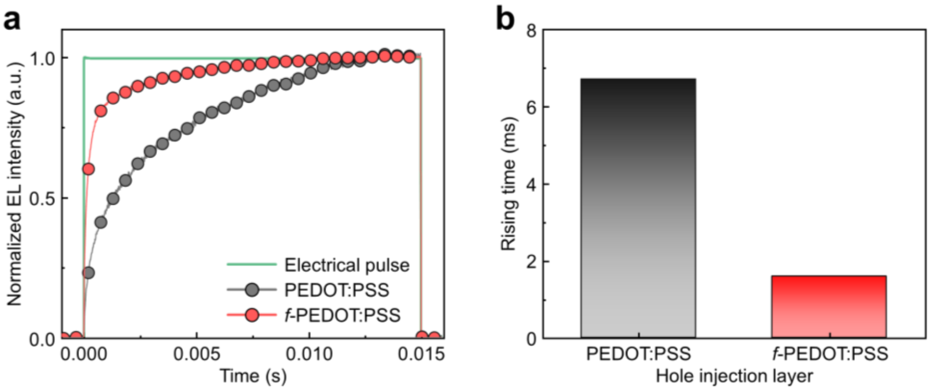

Figure S32. a) TREL characteristics of LEDs fabricated with as-synthesized NCs using either PEDOT:PSS or *f*-PEDOT:PSS as hole-injection layer. b) Rising times corresponded to TREL data, defined as the time required for the EL intensity to reach 80% of its maximum value.

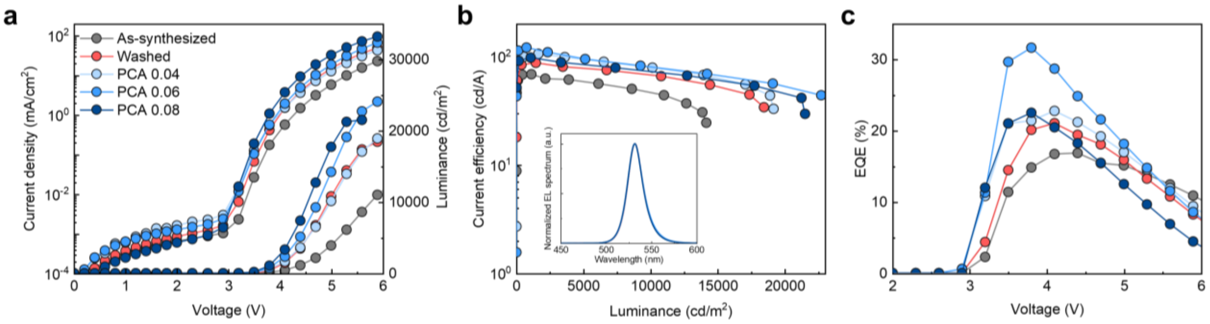

Figure S33. a) Current density and luminance versus voltage characteristics, b) current efficiency versus luminance (Inset: EL spectrum that use the as-synthesized, washed, and *LE* NCs with varying PCA concentrations as an EML), and c) EQE versus voltage characteristics.

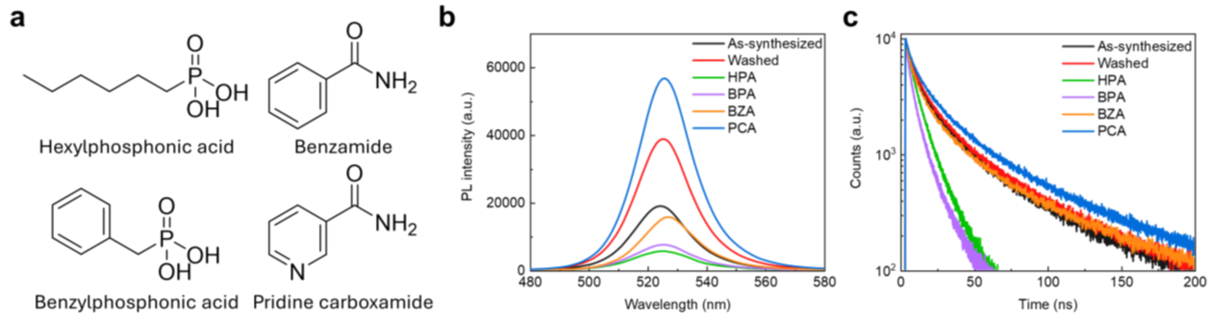

Figure S34. a) Various ligand molecule structures. b) Steady-state PL spectra, and c) TRPL of the colloidal NC solutions treated with various ligand molecules.

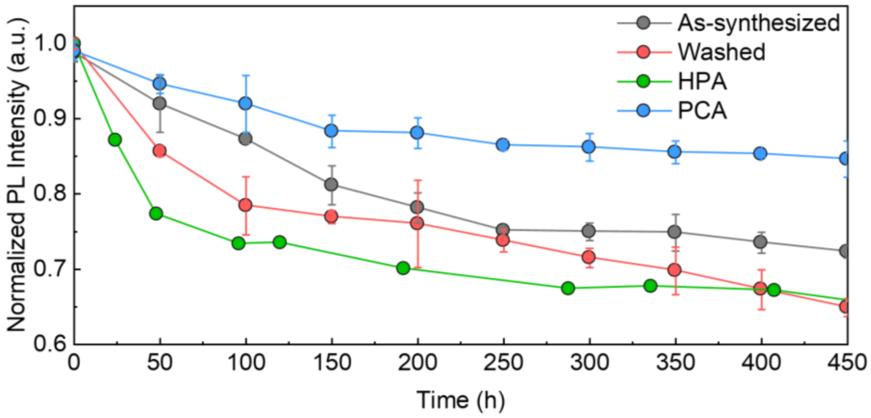

Figure S35. Evolution of steady-state PL intensity versus time under ambient conditions at room temperature.

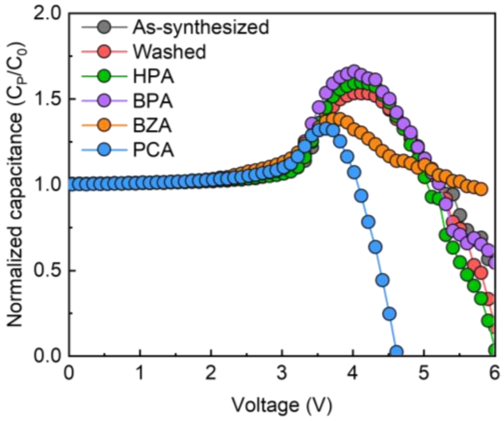

Figure S36. Normalized capacitance versus voltage characteristics of LEDs employing NCs treated with various ligand molecules.

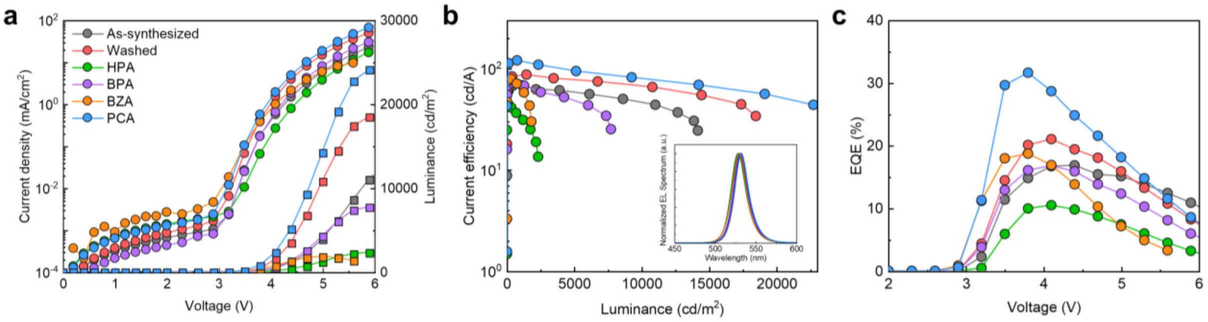

Figure S37. a) Current density and luminance versus voltage characteristics, b) current efficiency versus luminance characteristics (Inset: EL spectrum of LEDs), and c) EQE versus voltage characteristics of LEDs that use NCs treated with various ligand molecules.

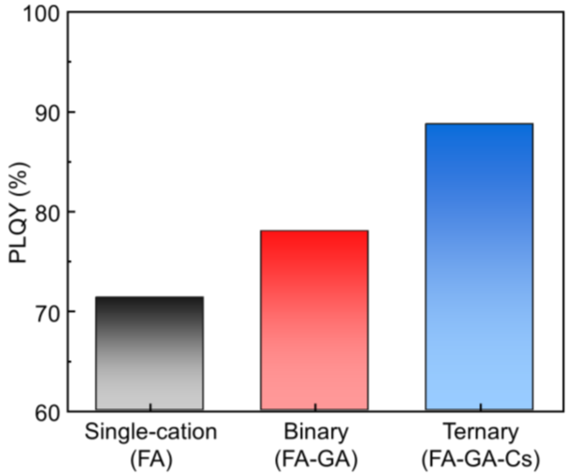

Figure S38. PLQYs of the single-cation (FA), binary-cations (FA-GA), and ternary (FA-GA-Cs)-cations composition NC solutions.

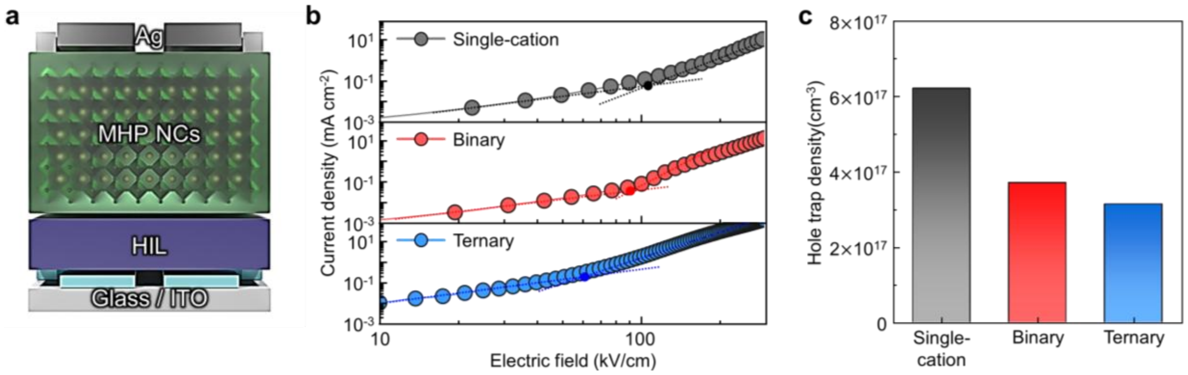

Figure S39. a) Illustration of fabricated HODs architecture. b) Current density versus electric field characteristics of HODs, and c) hole trap densities extracted from the trap-filled limited regime of HODs with the single-cation, binary-, ternary-composition NCs.

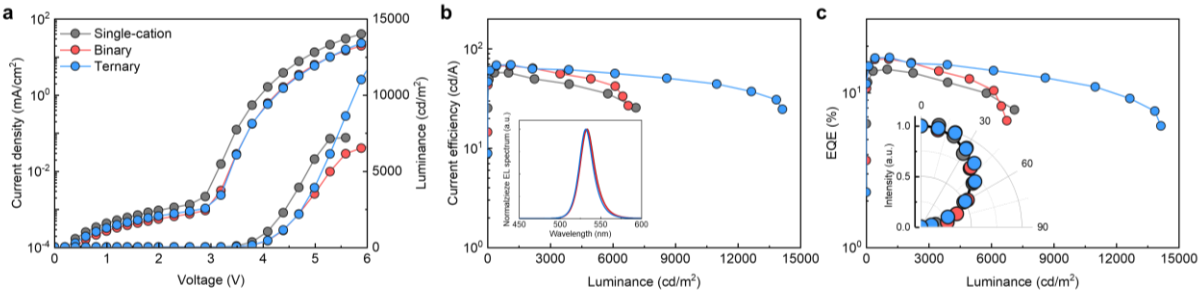

Figure S40. a) Current density and luminance versus voltage, b) current efficiency versus luminance (Inset: EL spectrum of LEDs), and c) EQE versus voltage of LEDs that use the single-cation, binary-, ternary-composition NCs as an EML (Inset: viewing angle-dependent EL emission profile used for EQE calculation).

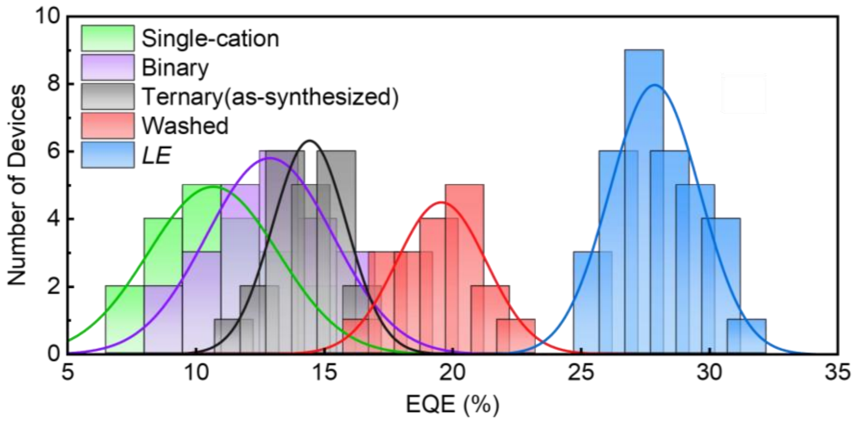

Figure S41. EQE histogram of LEDs that use the single-cation, binary-, ternary- (as-synthesized) composition NCs, and washed, *LE* NCs as an EML.

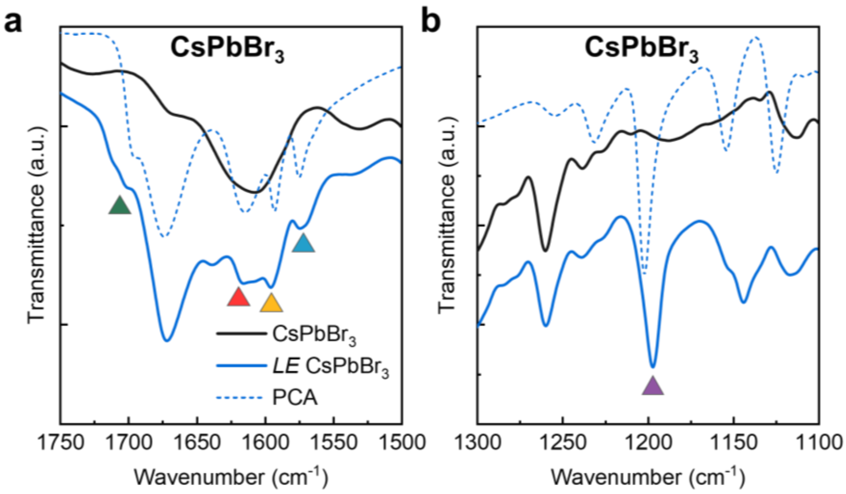

Figure S42. FTIR spectra of PCA and MHP NCs for a) C=O stretching, N-H bending, and C=N stretching, and b) C-N stretching for the as-synthesized and *LE* $CsPbBr_3$ NCs.

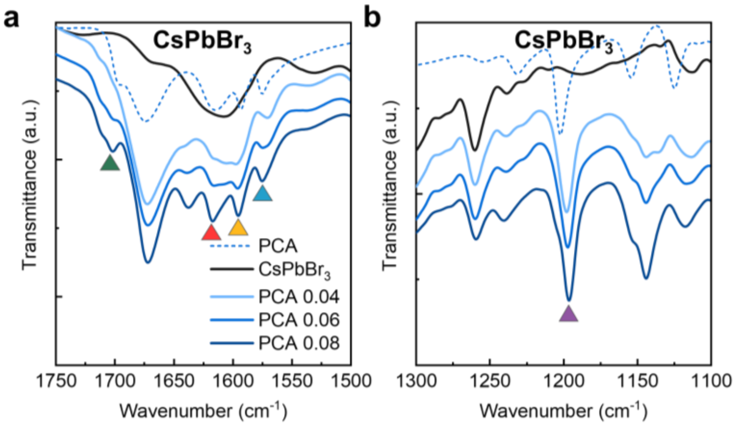

Figure S43. FTIR spectra of PCA and MHP NCs with varying PCA concentrations for a) C=O stretching, N-H bending, and C=N stretching, and b) C-N stretching for as-synthesized and *LE* $CsPbBr_3$ NCs.

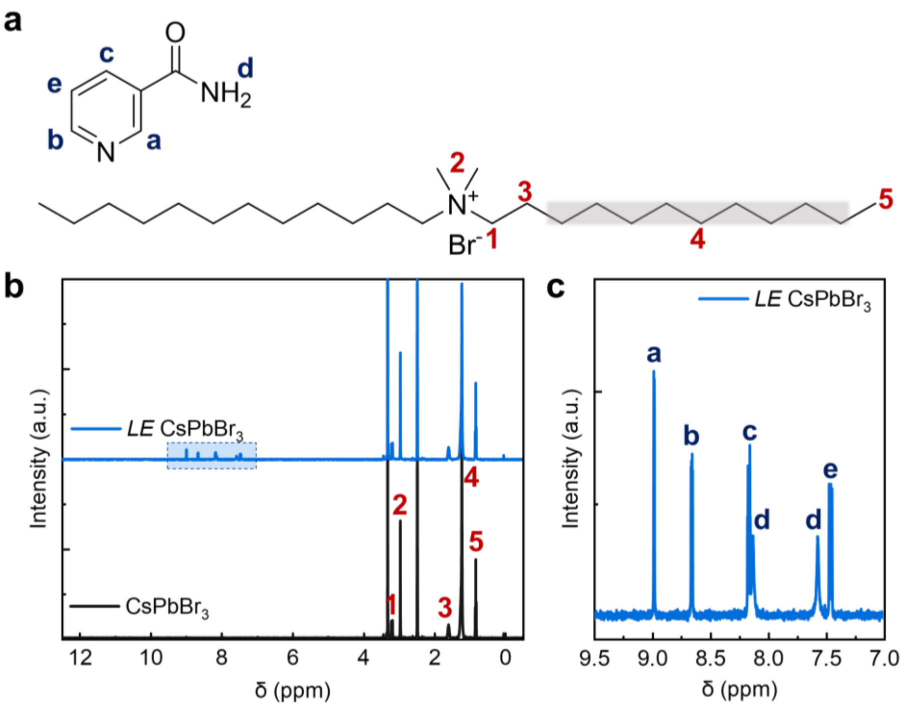

Figure S44. a) Molecular structure of PCA and DDAB, and b) ^{1}H NMR spectra of as-synthesized and *LE* $CsPbBr_3$ NCs. c) ^{1}H NMR spectra of *LE* $CsPbBr_3$ NCs in 9.5-7.0 ppm region.

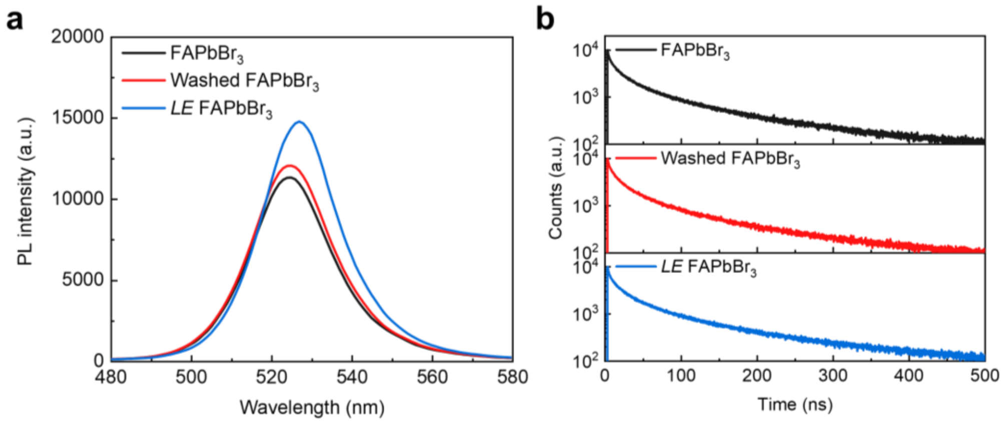

Figure S45. a) Steady-state PL spectra, and b) TRPL of the FA-based colloidal NC solutions.

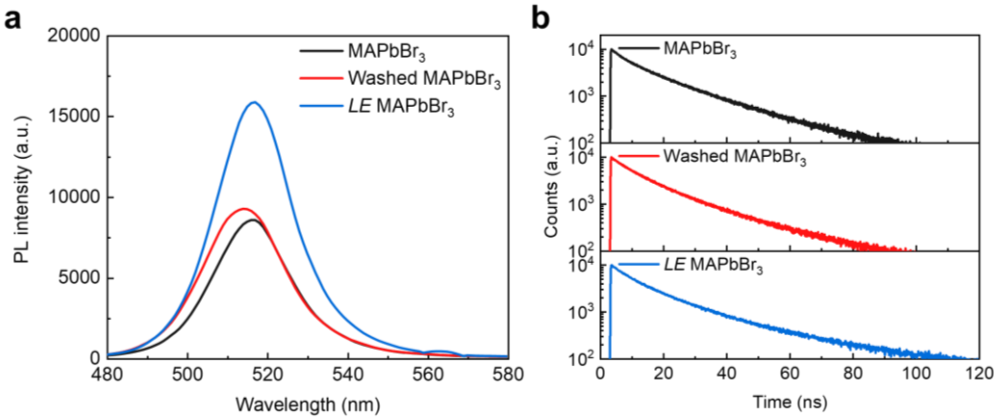

Figure S46. a) Steady-state PL spectra, and b) TRPL of the MA-based colloidal NC solutions.

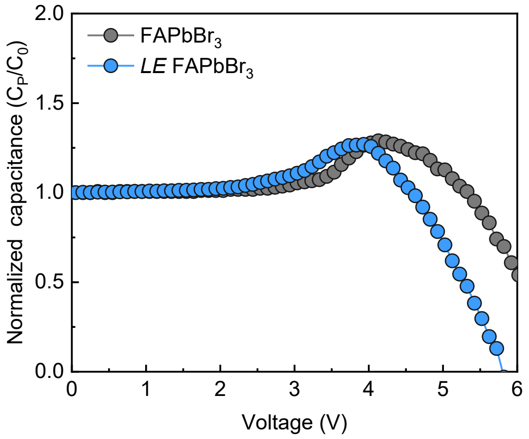

Figure S47. Normalized capacitance versus voltage characteristics of LEDs that use the FA-based NCs as an EML.

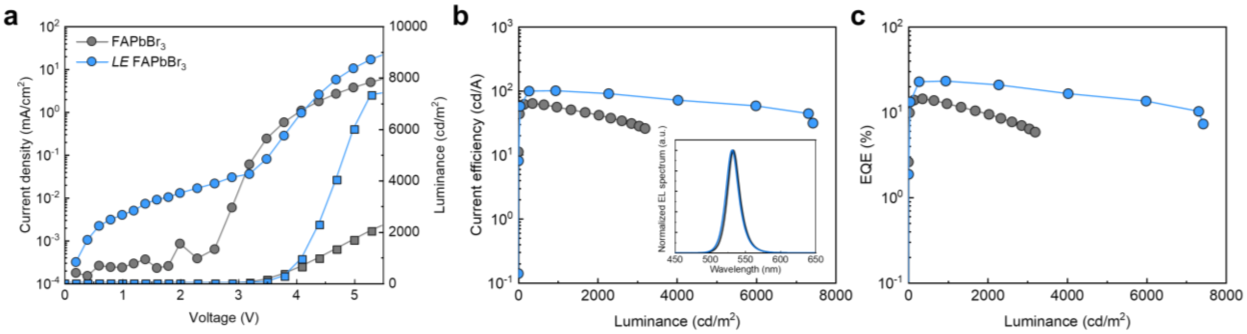

Figure S48. a) Current density and luminance versus voltage characteristics, b) current efficiency versus luminance characteristics (Inset: EL spectrum of LEDs), and c) EQE versus luminance characteristics of LEDs that use the FA-based NCs as an EML.

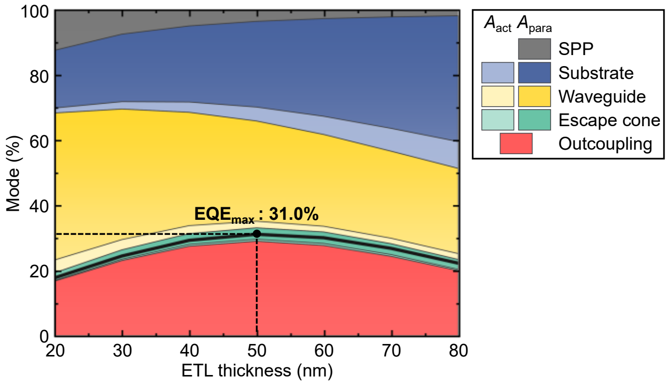

Figure S49. Optical simulation of relative mode fraction in PEDOT:PSS-based MHP NC LED as a function of electron transport layer thickness.

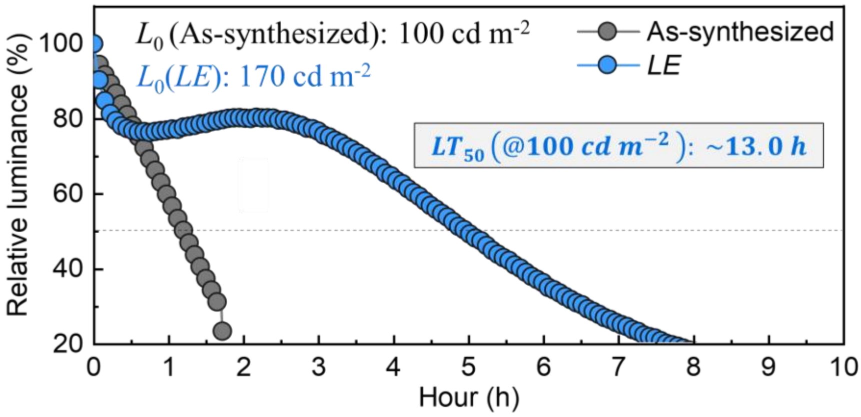

Figure S50. Evolution of luminance according to time passed at a constant current.

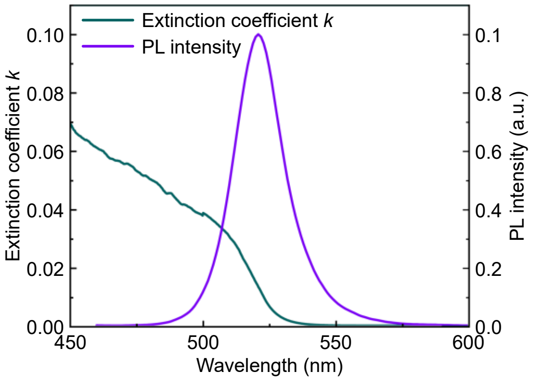

Figure S51. Extinction coefficient *k* spectra extracted from the experimentally measured absorption coefficient and PL spectra of *LE* NC.

Supporting Tables

Table S1. Lifetime parameters in TRPL decay of MHP NC films

	**As-synthesized**	**Washed**	***LE***
**A_1**	9,953.57	8,487.16	5,597.7
**Proportion (%)**	63.44	58.98	55.44
**τ_1 (ns)**	4.15	4.78	7.6
**A_2**	5,735.06	5,903.28	4,498.32
**Proportion (%)**	36.56	41.02	44.56
**τ_2 (ns)**	23.61	26.26	32.63
**τ_{avg} (ns)**	11.23	13.59	18.76

Table S2. Lifetime parameters in TRPL decay of colloidal MHP NC solutions.

	**As-synthesized**	**Washed**	***LE***
**A_1**	10,900.87	9,953.76	8,875.91
**Proportion (%)**	66.04	63.44	59.97
**τ_1 (ns)**	3.96	4.15	4.58
**A_2**	5,605.59	5,736.22	5,923.42
**Proportion (%)**	33.96	36.56	40.02
**τ_2 (ns)**	22.72	23.61	26.54
**τ_{avg} (ns)**	10.33	11.26	13.37

Table S3. Lifetime parameters in TRPL decay of NC solutions treated with various ligands.

	**As-synthesized**	**Washed**	**HPA**	**BPA**	**BZA**	**PCA**
**A_1**	10,900.87	9,953.76	12,155.79	19,049.82	11,474.09	8,875.91
**Proportion (%)**	66.04	63.44	70.05	79.58	74.68	59.97
**τ_1 (ns)**	3.96	4.15	4.63	2.94	4.94	4.58
**A_2**	5,605.59	5,736.22	5,198.30	4,888.52	3,891.07	5,923.42
**Proportion (%)**	33.96	36.56	29.95	20.42	25.32	40.02
**τ_2 (ns)**	22.72	23.61	13.99	11.71	31.57	26.54
**τ_{avg} (ns)**	10.33	11.26	7.43	4.73	11.70	13.37

Table S4. Lifetime parameters in TRPL decay of the FA-based NC colloidal solutions.

	**$FAPbBr_3$**	**Washed $FAPbBr_3$**	***LE* $FAPbBr_3$**
**A_1**	8,869.04	8,495.35	8,656.30
**Proportion (%)**	67.25	66.83	66.55
**τ_1 (ns)**	4.91	5.49	5.08
**A_2**	4,318.94	4,216.47	4,350.14
**Proportion (%)**	32.75	33.17	33.45
**τ_2 (ns)**	41.76	41.45	42.89
**τ_{avg} (ns)**	16.97	17.42	17.73

Table S5. Lifetime parameters in TRPL decay of the MA-based NC colloidal solutions.

	**$MAPbBr_3$**	**Washed $MAPbBr_3$**	***LE* $MAPbBr_3$**
**A_1**	8,503.06	9,369.36	9,715.24
**Proportion (%)**	55.51	66.69	67.45
**τ_1 (ns)**	4.74	6.98	6.65
**A_2**	6,816.05	4,678.72	4,688.82
**Proportion (%)**	44.49	33.31	32.55
**τ_2 (ns)**	18.78	20.88	22.29
**τ_{avg} (ns)**	10.99	11.61	11.74

Supporting References

[S1] Y. H. Kim, S. Kim, A. Kakekhani, J. Park, J. Park, Y. H. Lee, H. Xu, S. Nagane, R. B. Wexler, D. H. Kim, S. H. Jo, L. Martínez-Sarti, P. Tan, A. Sadhanala, G. S. Park, Y. W. Kim, B. Hu, H. J. Bolink, S. Yoo, R. H. Friend, A. M. Rappe, T. W. Lee, *Nat. Photon.* **2021**, *15*, 148-155.

[S2] N. Fiuza-Maneiro, K. Sun, I. López-Fernández, S. Gómez-Graña, P. Müller-Buschbaum, L. Polavarapu, *ACS Energy Lett.* **2023**, *8*, 1152-1191.

[S3] F. Haydous, J. M. Gardner, U. B. Cappel, *J. Mater. Chem. A* **2021**, *9*, 23419–23443.

[S4] D. Y. Kwok, A. W. Neumann, *Adv. Colloid. Interface Sci.* **1999**, *81*, 167.

[S5] P. Peumans, A. Yakimov, S. R. Forrest, *J. Appl. Phys.* **2003**, *93*, 3693-3723.

[S6] T. H. Han, M. R. Choi, S. H. Woo, S. Y. Min, C. L. Lee, T. W. Lee, Adv. Mater. **2012**, 24, 1487-1493.

[S7] L. Canil, T. Cramer, B. Fraboni, D. Ricciarelli, D. Meggiolaro, A. Singh, M. Liu, M. Rusu, C. M. Wolff, N. Phung, Q. Wang, D. Neher, T. Unold, P. Vivo, A. Gagliardi, F. De Angelis, A. Abate, *Energy Environ. Sci.* **2021**, *14*, 1429-1438.

[S8] T. H. Han, W. Song, T. W. Lee, *ACS Appl. Mater. Interfaces* **2015**, *7*, 3117-3125.

[S9] T. H. Han, Y. H. Kim, M. H. Kim, W. Song, T. W. Lee, *ACS Appl. Mater. Interfaces* **2016**, *8*, 6152-6163.

[S10] Y. Shen, X. M. Hu, M. L. Guo, Y. Q. Li, J. X. Tang, *J. Phys. Chem. Lett.* **2024**, *15*, 7916-7923.

[S11] X. Xue, B. Zhu, Z. Kang, X. Chi, H. Zhang, A. Tang, W. Ji, *Laser Photon. Rev.* **2024**, *19*, 2401166.

[S12] E. Cha, J. Jeon, H. W. Kim, H. U. Lee, J. Y. Woo, J.-S. Yeo, H. Bin Kwon, S. B. Cho, T.-H. Han, *Adv. Funct. Mater.* **2025**, e08146.

[S13] P. M. Hangoma, E. Yang, F. Liu, D. Kim, C.-M. Oh, H. S. Yang, I.-W. Hwang, B. R. Lee, S. H. Park, P. M. Hangoma, E. Yang, F. Liu, S. H. Park, D. Kim, H. S. Yang, B. R. Lee, C.-M. Oh, I.-W. Hwang, *Small* **2025**, 2407519.

[S14] D. Lee, S. M. Lee, A. Y. Lee, J. Kim, J. Lee, D. H. Kwon, J. Han, Y. W. Noh, W. G. Shin, S. S. Choi, B. R. Lee, S. Lee, S. K. Kwak, M. H. Song, *ACS Energy Lett.* **2025**, *10*, 1411-1420.

[S15] Z. Ma, W. Zhou, D. Huang, Q. Liu, Z. Xiao, H. Jiang, Z. Yang, W. Zhang, Y. Huang, *ACS Appl. Mater. Interfaces* **2020**, *12*, 52500–52508.

[S16] E. Uspenskaya, T. Pleteneva, A. Syroeshkin, I. Kasymova, N. Zakharova, *BIO Web Conf.* **2020**, *22*, 01005.

[S17] H. Lv, N. Gao, Q. Zhou, Y. Wang, G. Ling, P. Zhang, *Adv. Healthc. Mater.* **2023**, *12*, 2203295.

[S18] S. Mallick, M. Murugesan, *Phys. Chem. Liq.* **2021**, *59*, 345-357.

[S19] J. Wang, M. Li, B. Cai, H. Ren, W. Fan, L. Xu, J. Yao, S. Wang, J. Song, *Angew. Chem. Int. Ed.* **2024**, *63*, e202410689
